# Lipidomic and Metabolomic Signature of Progression of Chronic Kidney Disease in Patients with Severe Obesity

**DOI:** 10.3390/metabo11120836

**Published:** 2021-12-03

**Authors:** Borja Lanzon, Marina Martin-Taboada, Victor Castro-Alves, Rocio Vila-Bedmar, Ignacio González de Pablos, Daniel Duberg, Pilar Gomez, Elias Rodriguez, Matej Orešič, Tuulia Hyötyläinen, Enrique Morales, Francisco J. Ruperez, Gema Medina-Gomez

**Affiliations:** 1 LIPOBETA Group, Department Basic Sciences of Health, Faculty of Sciences of Health, Universidad Rey Juan Carlos, 28922 Alcorcón, Spain; borja.lanzon@urjc.es (B.L.); marina.martin@urjc.es (M.M.-T.); rocio.vila@urjc.es (R.V.-B.); 2 School of Science and Technology, Örebro University, 702 81 Örebro, Sweden; Victor.Castro-Alves@oru.se (V.C.-A.); Daniel.Duberg@oru.se (D.D.); Tuulia.Hyotylainen@oru.se (T.H.); 3 Department of Nephrology, University Hospital 12 de Octubre, 28041 Madrid, Spain; igp.snurse@gmail.com (I.G.d.P.); emorales@salud.madrid.org (E.M.); 4 Department of Surgery, Hospital Universitario 12 de Octubre, 28041 Madrid, Spain; pilgom@hotmail.com (P.G.); elias.rodriguezcue@salud.madrid.org (E.R.); 5 School of Medical Sciences, Örebro University, 702 81 Örebro, Sweden; Matej.Oresic@oru.se; 6 Turku Bioscience, University of Turku and Åbo Akademi University, 20520 Turku, Finland; 7 Research Institute of University Hospital 12 de Octubre (imas12), 28041 Madrid, Spain; 8 Department of Medicine, Complutense University of Madrid, 28041 Madrid, Spain; 9 Centro de Metabolómica y Bioanálisis (CEMBIO), Universidad San Pablo-CEU, CEU Universities, 28668 Boadilla del Monte, Spain; ruperez@ceu.es; 10 LAFEMEX Laboratory, Área de Bioquímica y Biología Molecular, Departamento de Ciencias Básicas de la Salud, Facultad de Ciencias de la Salud, Universidad Rey Juan Carlos, 28922 Alcorcón, Spain

**Keywords:** severe obesity, CKD, lipidomics, metabolomics, bariatric surgery

## Abstract

Severe obesity is a major risk for chronic kidney disease (CKD). Early detection and careful monitoring of renal function are critical for the prevention of CKD during obesity, since biopsies are not performed in patients with CKD and diagnosis is dependent on the assessment of clinical parameters. To explore whether distinct lipid and metabolic signatures in obesity may signify early stages of pathogenesis toward CKD, liquid chromatography-mass spectrometry (LC-MS) and gas chromatography-high resolution accurate mass-mass spectrometry (GC-HRAM-MS) analyses were performed in the serum and the urine of severely obese patients with and without CKD. Moreover, the impact of bariatric surgery (BS) in lipid and metabolic signature was also studied, through LC-MS and GC-HRAM-MS analyses in the serum and urine of patients with severe obesity and CKD before and after undergoing BS. Regarding patients with severe obesity and CKD compared to severely obese patients without CKD, serum lipidome analysis revealed significant differences in lipid signature. Furthermore, serum metabolomics profile revealed significant changes in specific amino acids, with isoleucine and tyrosine, increased in CKD patients compared with patients without CKD. LC-MS and GC-HRAM-MS analysis in serum of patients with severe obesity and CKD after BS showed downregulation of levels of triglycerides (TGs) and diglycerides (DGs) as well as a decrease in branched-chain amino acid (BCAA), lysine, threonine, proline, and serine. In addition, BS removed most of the correlations in CKD patients against biochemical parameters related to kidney dysfunction. Concerning urine analysis, hippuric acid, valine and glutamine were significantly decreased in urine from CKD patients after surgery. Interestingly, bariatric surgery did not restore all the lipid species, some of them decreased, hence drawing attention to them as potential targets for early diagnosis or therapeutic intervention. Results obtained in this study would justify the use of comprehensive mass spectrometry-based lipidomics to measure other lipids aside from conventional lipid profiles and to validate possible early markers of risk of CKD in patients with severe obesity.

## 1. Introduction

According to World Health Organization, in 2016, over 55 million adults suffered from severe obesity supporting the current consideration of obesity as a global pandemic [1,2]. Obesity is the entry point for the development of different comorbidities, such as type 2 diabetes (T2D), dyslipidemia, or hypertension. Furthermore, obesity and diabetes are primary causes for the appearance and progression of chronic kidney disease (CKD) [3]. The deleterious effects of obesity on kidney function are associated with a decrease in the estimated glomerular filtration rate (eGFR) and a higher prevalence of albuminuria and proteinuria [4], among other alterations. However, there are no reliable markers of risk for early detection of CKD in patients with severe obesity. Moreover, biopsies are not performed in patients with CKD and diagnosis is dependent on the assessment of various clinical parameters [5]. All this importantly contributes to the fact that there is a high prevalence of undiagnosed patients with CKD, especially in those undiagnosed with other comorbidities [6]. Thus, it is imperative to find early markers in order to improve CKD diagnosis, so that these individuals may benefit from early interventions in order to prevent the development and/or progression of CKD.

Underlying biochemical activities undergoing in patients with severe obesity with existing CKD can be approached with metabolomics techniques offering knowledge in the underlying cellular mechanisms in the dynamic process of renal damage to the established disease [7]. Several authors have used this analytical approach to establish the alterations caused in the serum metabolomic signature in pathologies, such as obesity and CKD [8,9]. Moreover, there are no published studies that characterize the serum profile of patients with severe obesity and CKD to identify the concurrent differential factors in this context of metabolic impairment.

Different studies have highlighted the role of body weight loss in the improvement of renal function. In this regard, bariatric surgery (BS), has been accepted as the most effective option to lose weight. Published studies demonstrate that BS improves biochemical, structural, and ultrastructural measures of experimental diabetic kidney disease and interrupts the transcriptional program characteristic of progressive CKD [10]. The metabolomics approach has also been used to study the metabolic effects of BS [11]. However, to our knowledge, the relation between blood lipidomics, metabolomics, and the development of CKD has never been examined in individuals with morbid obesity.

The aim of this study is to analyze the existing alterations in CKD patients with severe obesity applying metabolomics and lipidomic techniques compared with patients without CKD. Further, this study has the advantage of also investigating the association of lipids and metabolites identified in serum and urine with the amelioration in kidney function obtained after one year of bariatric surgery in the same patients. Hence, this study opens a new avenue in the elucidation of new markers of risk for the diagnosis of CKD in patients with severe to morbid obesity, so that new strategies can be developed to prevent the development and/or progression of CKD in these patients.

## 2. Results

### 2.1. Impact on the Metabolomic and Lipidomic Fingerprint in Severe Obese Individuals with Chronic Kidney Disease

#### 2.1.1. Cohort Characterization: Body Weight and Biochemical Analyses

**Figure 1:**
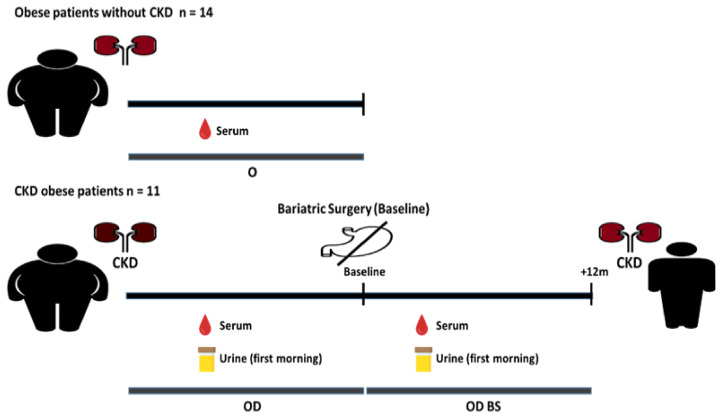
Sample collection scheme for CKD patients with severe obesity before and after surgery, and patients with severe obesity without CKD. Serum samples and urine samples from OD patients were collected before the bariatric surgery (baseline). Serum samples and urine from OD BS patients were collected after the clinical intervention. First-morning urine was collected from eight patients with CKD before and after the surgery. Serum samples were collected from O patients.

Eleven obese patients with CKD (OD) and fourteen obese patients without CKD (O) were screened and included in the study (Figure 1, study design). Body weights and biochemical measurements are shown in Table 1. The percentage of males was higher in OD than in O subjects. No significant differences were found in age, body weight and BMI between groups. Regarding the OD cohort, 63.63% were diabetic and followed a T2D treatment; 90.90% of OD were hypertensive and the whole OD cohort was treated for hypertension; 63.63% of OD were taking lipid-lowering drugs. Concerning O patients, 28.57% were diagnosed and treated for diabetes; 35.71% were hypertensive and were treated for hypertension; 14.28% were taking lipid-lowering drugs. Patients with kidney disease presented significantly higher glucose levels. Measurements of total circulating cholesterol and LDL did not show significant differences between groups. However, HDL levels were significantly lower in OD. Interestingly, OD patients presented TG levels significantly elevated than O patients. Kidney disease was diagnosed in OD patients by clinical criteria, with an increase of serum creatinine and uric acid in serum. eGFR measurement was remarkably lower in OD patients, however, levels of proteinuria and UACR (urinary albumin:creatinine ratio) were significantly higher in OD compared to O patients.

The possible differential contribution of taking lipid-lowering drugs in the lipidomic fingerprint (473 features obtained after the relative standard deviation, RSD, filtration) in OD patients was analyzed. The average of the total useful signal (TUS) obtained from CKD patients that were taking the drugs was compared with those that were not. The variation obtained in the average total useful signal (TUS) between both groups was 4.08% for the percentage of change and 0.06 for the Log2FC. The degree of changes presented by OD patients with lipid-lowering drugs vs. OD patients without lipid-lowering drugs showed that most of the features contained in the lipidomic fingerprint, 83.93% (397 out of 473), presented a distribution within the interval of −0.6 to 0.6 of the Log2FC, suggesting a minimal impact of lipid-lowering drugs in the lipidome signature in OD patients.

#### 2.1.2. De Novo Synthesis of Phospholipids and Fatty Acid Remodeling Were Significantly Increased in Patients with CKD

**Figure 2:**
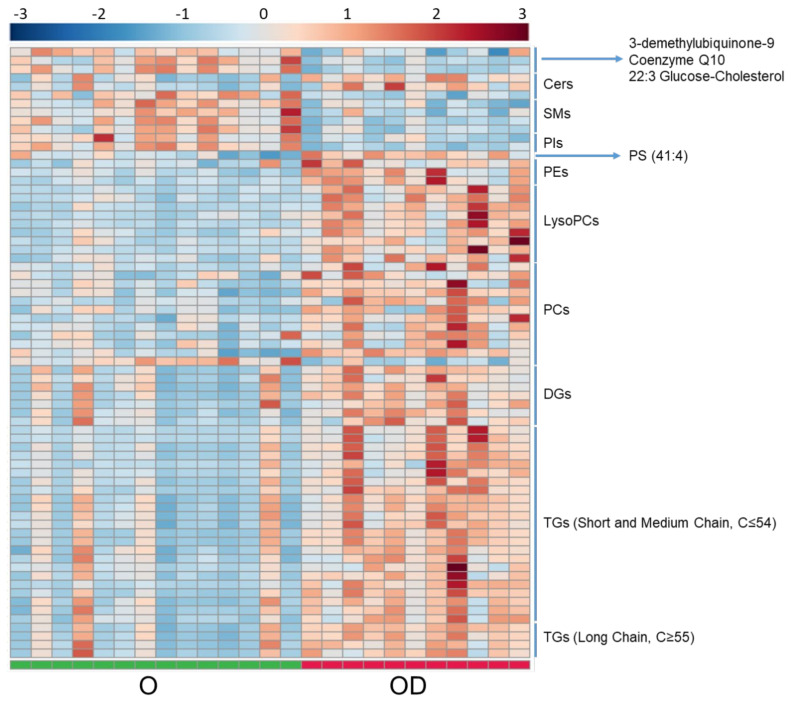
Heatmap for OD vs. O comparison in serum analyzed by LC-MS. Heatmap was generated with the peak intensities of the significant compounds found between OD ad O patients in the serum analyzed by LC-MS. OD patients (right) were represented with red color. O patients (left) were represented with green color. Abbreviations: TG, Triglycerides. DG, Diglycerides. PC, Phosphatidylcholine. LPC, Lysophosphatidylcholine. Cer, Ceramide. SM, Sphingomyelin. PI, Phosphatidylinositol. PE, Phosphatidylethanolamine. PS, Phosphatidylserine.

**Figure 3:**
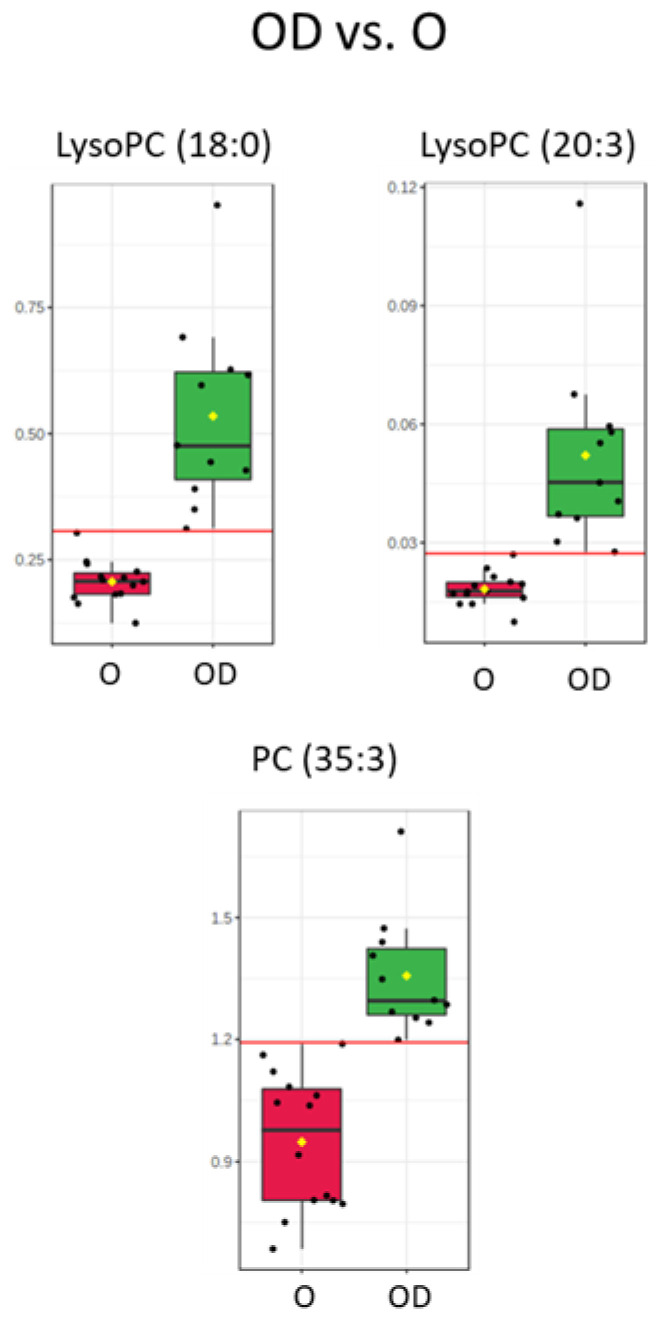
Box and whisker plots with the distribution of samples per group in features that presented an AUC value of one in the curve ROC analysis performed between OD vs. O patients. LysoPC (18:0), LysoPC (20:3) and PC (35:3) were obtained in the serum analyzed by LC-MS. Abbreviations: LysoPC, Lysophosphatidylcholine. PC, Phosphatidylcholine. O patients (left), red. OD patients (right), green.

Serum lipidome was analyzed by LC-MS in ion positive mode. After signal processing, data treatment and statistical analyses, 71 significant lipid compounds were annotated between OD and O subjects, and most of the lipid species were increased in OD patients (Figure 2, lipidomic heatmap for OD vs. O). In a close examination of the significant lipid compounds obtained in OD vs. O comparison (Table S4): 27 triglycerides (TGs) were found increased, of which, 23 were unsaturated. Eleven TG had short-chain (C ≤ 50), 12 medium-chain (C51 to C54) and five long-chain (C ≥ 55). Seven diglycerides (DGs) were found to be increased (from 32C to 36C) and six of these DGs were unsaturated; 11 significant phosphatidylcholines (PCs) were obtained (from 30C to 44C). In detail, nine significant increase PCs were unsaturated and just one, PC (42:8), presented a decreased fold change. Nine significantly increased lysophosphatidylcholines (LysoPCs) were obtained (from 14C to 20C) and unlike the rest of the lipid classes analyzed, there was a balance between saturated (5) and unsaturated (4) LysoPCs. LysoPC (18:0), LysoPC (20:3), and PC (35:3) reinforce the significance obtained in the statistical analysis with the results obtained in the ROC Curve test performed (Figure 3, box and whisker plots of features with an AUC value of one obtained in the ROC curve test in OD vs. O and Table S1) with AUC values of one. Shingomyelins (SMs) and phosphatidylinositols (PIs) were decreased in OD patients: four SMs were significant and were unsaturated, and only two PIs were found as significant and were unsaturated. Three ceramides (Cer) were found significant between OD and O patients. Ceramides (36:1) and (40:1) were upregulated, and Cer (44:1) was downregulated. Further, 12 significant lipids showed odd-numbered fatty acyl chains, specifically, one phosphatidylserine (PS), one LysoPC, four PCs, and six TGs presented odd fatty chains. It was interesting to note that three significant plasmalogens were obtained and they were also increased. Assessing the significant lipids obtained in OD vs. O comparison, 38% of lipids were TGs, 17% were PCs, 12% were LysoPCs and 10% were DGs. Links between features in OD vs. O comparison were included in the Supplementary Materials in the lipidomic pathway (Figure S1, Lipidomic pathway for OD vs. O); *p*-values and VIP values were included for each significant feature in Table S4. OPLS-DA model was included in Supplementary Materials Figure S6).

#### 2.1.3. Short Chain TG Showed a Negative Correlation with eGFR in OD Patients

All the significant lipid species obtained after the application of statistical tests were tested against biochemical parameters highly related to kidney dysfunction (Table 2). TGs, DGs, ceramides (Cer) and LysoPCs presented a high positive correlation with Glucose in O patients, however, those correlations were absent in OD patients. TGs were positively correlated with Cholesterol (short and medium-chain, eight TG were unsaturated), Uric acid (short-chain, two TG were unsaturated), Creatinine (short and medium-chain, six TG were unsaturated and three saturated), and Proteinuria (medium-chain, three TG were unsaturated) in OD patients. TGs were found negatively correlated to eGFR in OD patients. TGs found in this negative correlation were short-chain TG, of which, three were unsaturated. Seven LysoPC showed a strong positive correlation with LDL in OD patients (four LysoPC were saturated and three unsaturated). Ceramide (44:1) was negatively correlated to glucose and cholesterol in O patients (result showed in Table S3). Comprehensive correlation analysis can be found in Table S3.

#### 2.1.4. Essential Amino Acids Are Increased in Obese Patients with CKD

**Figure 4:**
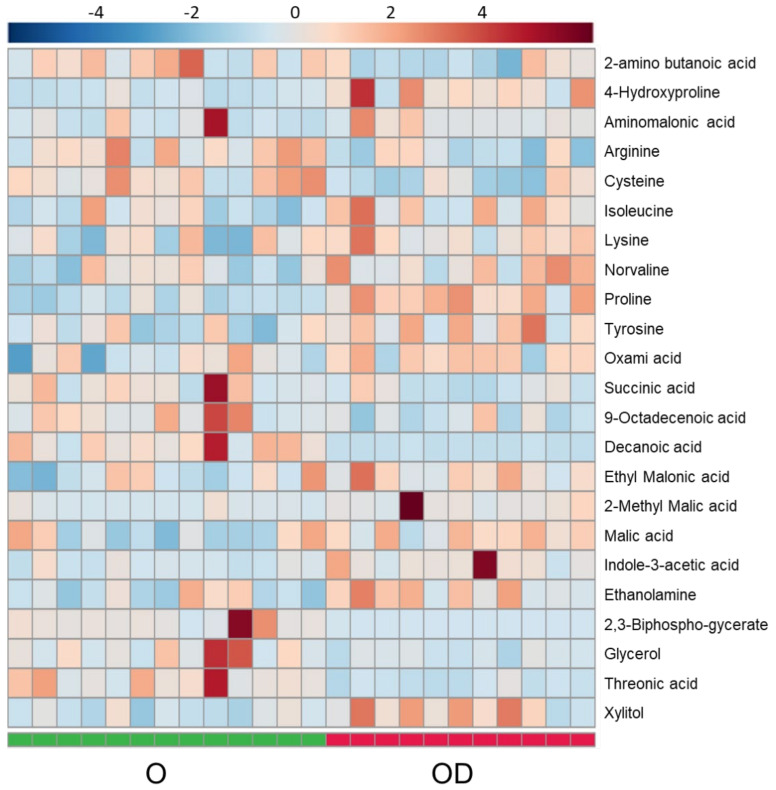
Heatmap for OD vs. O comparison in serum analyzed by GC-HRAM-MS. Heatmap was generated with the peak intensities of the significant compounds found between OD ad O patients in the serum analyzed by GC-HRAM-MS. OD patients (right) were represented with red color. O patients (left) were represented with green color.

Serum polar metabolomic profile was analyzed by GC-HRAM-MS; 23 significant polar metabolites were annotated between OD and O patients (Figure 4 and Table S4). Essential amino acids were mostly increased in OD patients and isoleucine and lysine were found significant. Assessing conditional amino acids, the trend was mixed with a significant increase in 4-hydroxyproline, proline and tyrosine, and a significant decrease in cysteine and arginine in CKD patients. In the TCA cycle, malate and succinate were significantly increased and decreased, respectively. Links between features in OD vs. O comparison were included in Supplementary Materials in the amino acid pathway (Figure S2, Amino acid pathway for OD vs. O). *p*-values and VIP values were included for each significant feature in Table S4. OPLS-DA model was included in Supplementary Materials Figure S7).

### 2.2. Bariatric Surgery Improves the Serum Lipidomic Profile and Metabolomic Fingerprint in Obese Patients with CKD

The same CKD patients with severe obesity analyzed previously (OD patients) underwent BS (OD BS patients) and samples of serum and urine were collected 12 months after surgery. As previously described by Morales et al. [12], CKD patients with severe obesity after bariatric surgery showed a drastic weight reduction with a significant decrease in proteinuria, albuminuria, uric acid measurements and an improvement in glomerular hyperfiltration and HDL levels (Figure 1). Weight loss after BS induces a triglyceride decrease in parallel with a decrease in adipokines and pro-inflammatory and pro-fibrotic parameters [12]. The possible differential contribution of the method used in the bariatric surgery in the lipidome from patients who underwent roux-en-Y gastric bypass and patients who underwent sleeve gastrectomy was analyzed. The average of the total useful signal (TUS) obtained from patients under the two different methods of surgery was 16.39% for the percentage of change and 0.26 for the Log2FC (Data not shown). The degree of changes presented by patients who underwent gastric bypass vs. sleeve gastrectomy showed that most of the features, 90.69%, presented a distribution within the interval 0.8 to 0.8 of the Log2FC suggesting that the different bariatric procedures did not introduce a remarkable variation in patients’ lipidome.

**Figure 5:**
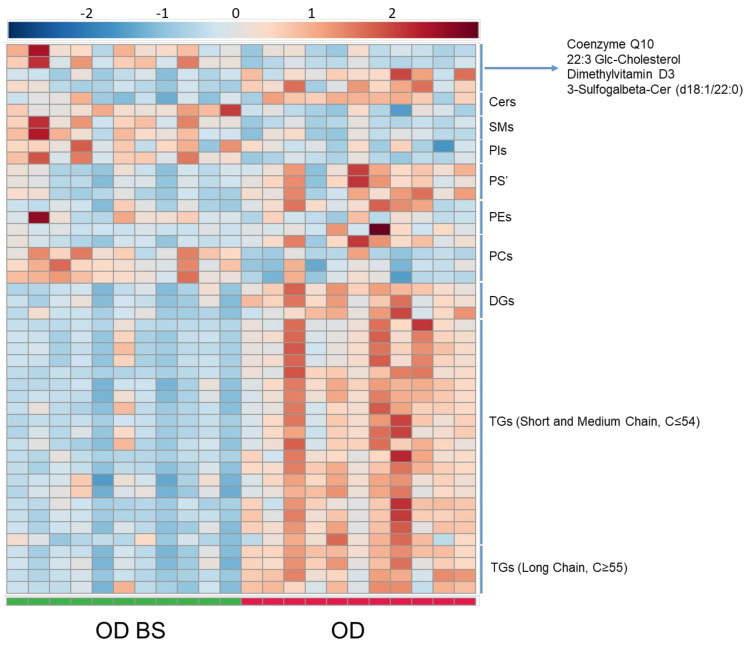
Heatmap for OD BS vs. OD comparison in serum analyzed by LC-MS. Heatmap was generated with the peak intensities of the significant compounds found between OD BS and OD patients in the serum analyzed by LC-MS. OD patients (right) were represented with red color. OD BS patients (left) were represented with green color. Abbreviations: TG, Triglycerides. DG, Diglycerides. PC, Phosphatidylcholine. Cer, Ceramide. SM, Sphingomyelin. PI, Phosphatidylinositol. PE, Phosphatidylethanolamine. PS, Phosphatidylserine.

**Figure 6:**
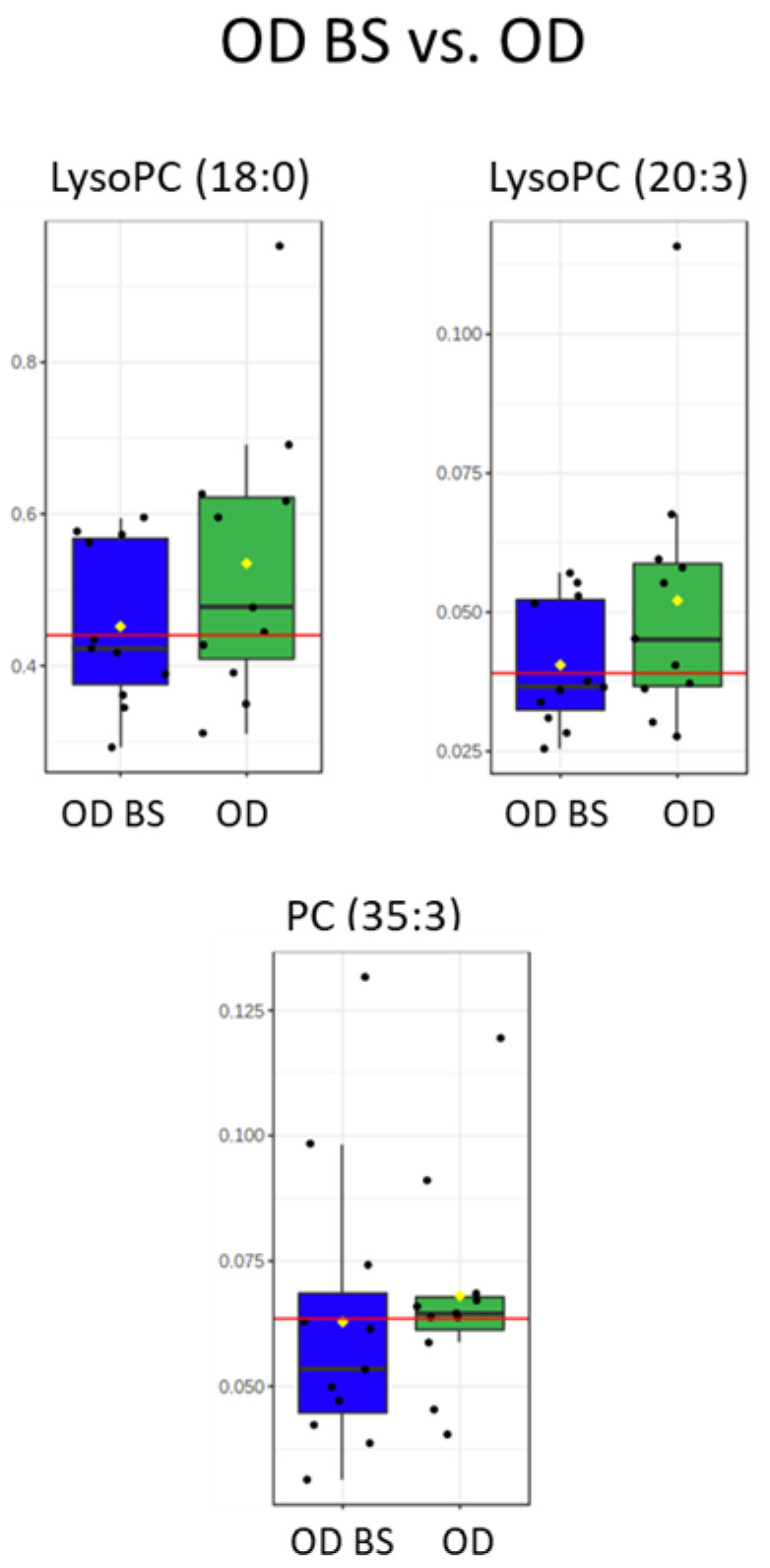
Box and whisker plots in OD BS and OD patients comparison with the distribution of samples that showed the best discriminatory capacity between OD and O patients in the ROC Curve Test: LysoPC (18:0), LysoPC (20:3) and PC (35:3). Abbreviations: LysoPC, Lysophosphatidylcholine. PC, Phosphatidylcholine. OD BS patients (left), blue. OD patients (right), green.

Lipid signatures were also analyzed in serum by LC-MS in these patients after 12 months of BS. The BS promoted differentiation in the lipidome and 46 significant lipids were annotated between these patients before and after surgery (Figure 5). Significant lipid compounds were generally decreased in OD BS. Analyzing the significant features obtained in OD BS vs. OD comparison (Table S4): 23 TGs were found to decrease and 20 of these TGs were unsaturated. Eight TGs had short-chain, 11 medium-chain, and four long-chain. Three DGs were decreased, of which, two were unsaturated. Regarding PCs, just three were found significant, two of them, PC (39:0) and PC (44:5) were increased and only one, PC (36:3) was decreased. Two decreased and unsaturated phosphatidylethanolamines (PEs) were found in the lipidomic analysis. Furthermore, three unsaturated PSs were decreased between OD BS vs. OD patients. Two PIs, two SMs, and two plasmalogens were increased and were also unsaturated. Despite all these changes in different lipid species, we did not find significant differences in glycerophosphocholines, mainly LysoPCs and most of the PCs, between OD BS and OD patients (Table S4). LysoPC (18:0), LysoPC (20:3) and PC (35:3), the features that presented the best discriminatory capacity between OD and O patients, did not show significant differences and presented a similar distribution between OD BS and OD patients (Figure 6). Assessing the significant lipids obtained in OD BS vs. OD comparison, 50% of lipids were TGs, 9% were PCs, around 6–7% were PEs, PS’ and DGs. Links between features in OD BS vs. OD comparison were included in Supplementary Materials in the lipidomic pathway (Figure S3, Lipidomic pathway for OD BS vs. OD). *p*-values and VIP values were included for each significant feature in Table S4. OPLS-DA model was included in Supplementary Materials Figure S8).

#### 2.2.1. Diglycerides and Medium-Chain Triglycerides Presented a Positive Correlation with Uric Acid in Obese Patients with CKD after Surgery

All the significant lipid species obtained after the application of statistical tests were tested against biochemical parameters highly related to kidney dysfunction (Table 3). Initially, while TG and DG were the species correlated with renal function markers in OD, after clinical intervention those correlations with creatinine, eGFR and proteinuria were lost. Bariatric surgery specifically modified the correlation presented with uric acid adding DG to the correlation and medium-chain TG, instead of the short-chain TG obtained in OD patients. Of note, the lack of correlations with UACR and glucose was present in patients before and after clinical intervention. Comprehensive correlation analysis results are included in Table S3.

**Figure 7:**
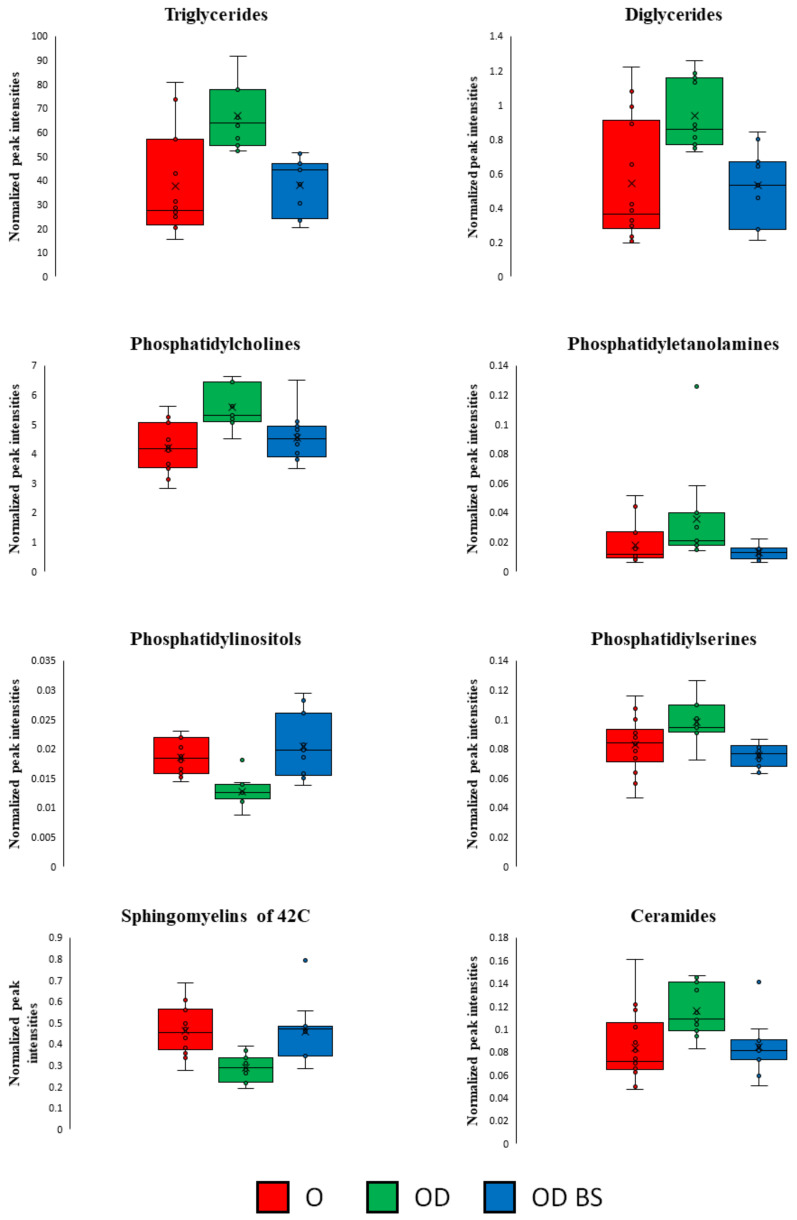
Representations of the sums of the intensity peaks of each lipid subclass per group of patients with box and whisker plots. Included all the significant features obtained in OD vs. O and OD BS vs. OD comparisons in the LC-MS analysis performed in serum. Axis Y: sums of the normalized peak intensities (areas) showed. OD BS patients presented a similar lipidomic profile than O patients. Red, O patients; Green, OD patients; Blue, OD BS patients.

Clinical intervention in OD BS patients produced changes in their lipidome that allowed them to reduce the differences compared to O patients at the lipidomic level. This behavior is supported by the similarity existing between OD BS and O samples in the PCA analysis (Figure S5). Furthermore, intensity peaks represented in box and whisker plots for TGs, DGs, PCs, PEs, PIs, PSs, Cers and SMs of 42C presented similar levels between OD BS and O patients (Figure 7).

#### 2.2.2. Isoleucine and Proline Decreased in the Serum of CKD Patients with Obesity after Bariatric Surgery

**Figure 8:**
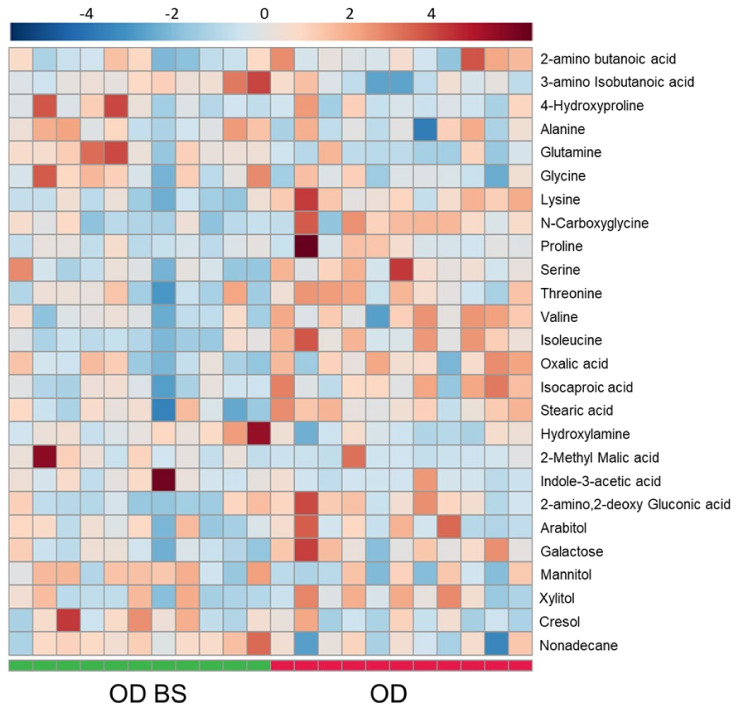
Heatmap for OD BS vs. OD comparison in serum analyzed by GC-HRAM-MS. Heatmap was generated with the peak intensities of the significant compounds found between OD BS and OD patients in the serum analyzed by LC-MS. OD patients (right) were represented with red color. OD BS patients (left) were represented with green color.

Polar metabolomics profile in serum was analyzed by GC-HRAM-MS; 28 significant polar metabolites were annotated between CKD subjects with obesity before and after bariatric surgery (Figure 8 and Table S4). Interestingly, four essential amino acids (isoleucine, lysine, threonine, and valine) and three conditional amino acids (proline, serine and tyrosine) were significantly downregulated. On the contrary, just two conditional amino acids, glutamine and glycine, were significantly upregulated after bariatric surgery. Furthermore, alanine, a non-essential amino acid, was significantly increased in OD BS. Links between features in OD BS vs. OD comparison were included in Supplementary Materials in the amino acid pathway (Figure S4. Amino acid pathway for OD BS vs. OD). *p*-values and VIP values were included for each significant feature in Table S4. OPLS-DA model was included in Supplementary Materials Figure S9).

### 2.3. Bariatric Surgery Decreased Levels of Valine and Glutamine in Urine from Patients with CKD

The polar metabolite fingerprint in urine between obese patients with CKD before and after surgery was analyzed by GC-HRAM-MS. Fifteen significant polar metabolites were annotated in these patients after surgery (Table S4). Valine and glutamine were significantly decreased. Hippuric acid showed significantly higher levels after surgery. *p*-values and VIP values were included for each significant feature in Table S4. OPLS-DA model was included in Supplementary Materials Figure S10).

## 3. Discussion

The present study reflects the impact of renal damage in patients with severe obesity using a comprehensive metabolomic point of view. To our knowledge, there are no published studies that cover this combination of factors of severe obesity and CKD with these metabolomics approaches in serum and urine. The analysis of the serum metabolome by LC-MS and GC-HRAM-MS revealed that CKD patients with severe obesity presented a significant increase in the circulating levels of different TGs, DGs, PEs, PCs, LysoPCs, suggesting a distinguishing signature for the lipidome in these patients with renal damage. Our results expose that morbidly obese patients with CKD undergoing bariatric surgery showed a decreased neutral lipids fingerprint associated with a significant decrease in proteinuria and renal function as well as control of metabolic parameters. We also identified a polar metabolic fingerprint in these patients associated with a decrease in the essential amino acids in serum and urine after bariatric surgery.

The intricate link between obesity and CKD is not completely decoded [13], but obesity is a significant risk factor for the development and progression of CKD [14], even individuals with an elevated BMI have a greater risk of developing proteinuria [15]. Importantly, CKD should be considered as a complication of overweight and obesity, regardless of whether the association was independent or through the influence of other factors, such as diabetes, hypertension, cardiovascular disease, metabolic syndrome and high fructose intake. However, the exact mechanisms underlying the association between obesity and CKD remain unclear [13]. A combination of hemodynamic and metabolic changes and renal lipotoxicity (excessive lipid deposition) may cause or aggravate CKD in obese individuals [15]. Firstly, the physiologic response of the kidney to obesity is mediated via an increase in renal plasma flow, glomerular filtration rate (GFR), filtration fraction, and proximal tubular reabsorption of sodium. Secondly, the altered production of adipokines and cytokines in an obesogenic state induces adaptive or maladaptive responses in renal cells against the mechanical forces of glomerular hyperfiltration. Many adipokines, including leptin, adiponectin, vascular endothelial growth factor, angiopoietins, and resistin, play a role in extracellular matrix accumulation, leading to renal fibrosis [16]. And finally, abnormal lipid accumulation induces detrimental changes to renal lipid metabolism that induce insulin resistance, enhanced levels of inflammation, oxidative stress and endoplasmic reticulum (ER) stress [17].

It is well known that fat accumulation in the kidneys causes structural and functional changes in glomerular and tubular epithelial cells, leading to the development of obesity-related glomerulopathy (ORG), a secondary form of focal segmental glomerulosclerosis (FSGS). These changes in the setting of T2D irreparably damage the kidney and lead to the progression of end-stage renal disease (ESRD) [3]. However, not all patients with obesity develop CKD, and even the limited presence of full nephrotic syndrome in ORG can be clinically significant since a progressive increase in proteinuria may go unnoticed for years, leading to a late diagnosis of renal failure [3]. Therefore, it is extremely important to identify those who are at higher risk. Our hypothesis is that a comprehensive lipidome analysis is crucial to the identification of potential markers of risk of CKD during obesity and elucidation of the mechanistic basis.

In our study, proteinuria and eGFR were the primary clinical parameters (markers of risk of CKD) used for the diagnosis of CKD in patients with severe obesity. Furthermore, the OD group comprised a higher number of hypertensive and diabetic subjects than the O group, which is reflected in lower values of HDL, and higher levels of TG and UACR on average in the OD group. Therefore, OD patients presented a worse metabolic condition than O patients. There were no significant differences in total cholesterol in OD vs. O comparison, associated with the fact that 63.6% of OD patients were taking lipid-lowering drugs. Moreover, it is important to consider in our study that lipid-lowering drugs homogenized the lipidomic profile of OD patients with a similar profile to OD patients that were not under the treatment. However, TGs were found to significantly increase in patients with severe obesity. Hypertriglyceridemia is a well-known condition of obese and CKD patients. Increased deposition of TGs in the kidneys has been associated with glomerular hyperfiltration, albuminuria and the increase of proinflammatory and proangiogenic cytokines [15]. Within the renal glomerular cells, specifically in podocytes, mesangial cells and proximal tubules, ectopic TGs accumulation leads to local inflammation, increased nitric oxide (NO) generation, mitochondrial dysfunctions and eventual fibrosis [18]. Therefore, high levels of serum TG (200–499 mg/dL) have been proposed as feasible predictors of hospitalization for new-onset kidney disease [19]. Furthermore, elevated levels of serum TG to HDL ratio have been associated with worsening of eGFR and CKD development in clinical studies [20]. HDL cholesterol levels were reduced in OD vs. O patients. It has been published that oxidant stress-mediated inflammation because of altered functionality of HDL contributes to the pathogenesis of kidney disease [21]. The presence of HDL dysfunctions could result in the subsequent reduction of anti-inflammatory and antioxidant properties in OD patients. However, we still need more data related to the HDL status issue to dampen the progression of CKD in obese patients.

Here, the aggravated hypertriglyceridemia condition of OD compared with O subjects is characterized by an increase of short and medium-chain TGs mainly, complemented by elevated levels of DGs. We came across strong positive correlations of TGs with the clinical parameter proteinuria in OD patients, not found in O subjects without renal disease. Moreover, the analysis demonstrated that eGFR was negatively correlated with TGs in OD patients. This points to a possible utility of TGs as a CKD progression marker of risk once CKD is established. In addition, the impact of deposition of saturated vs. unsaturated lipids in the progression of CKD has been discussed [22]. In this regard, the accumulation of ectopic lipid and lipid intermediates, like palmitate, ceramide, saturated NEFA, derived from other sources contribute significantly toward the onset and progression of CKD [23]. Although, in our study, we found positive correlations of short and medium-chain TGs with serum creatinine in both groups of patients, the saturated and unsaturated TG were correlated with this indirect parameter of renal function in OD patients but only unsaturated in O patients.

Studies of lipids in CKD, including End-stage renal disease (ESRD), have been limited to measures of conventional lipid profiles [24]. It is remarkable that in our study, simultaneous upregulated levels of PCs and LysoPCs became a differential marker of the concurrency of CKD in patients with severe obesity, suggesting that these lipids could be possible markers of risk of renal dysfunction in these patients. PCs and LysoPCs are typically upregulated during obesity [8] and serum LysoPCs and urine PCs have been proposed as CKD progression biomarkers [25,26]. Although the enzyme lecithin cholesterol ester transfer protein (LCAT) has been proposed as a unique tool to evaluate the impact of alterations in the HDL system on the progression of renal disease, LysoPCs concentration also depends on LCAT, which activity is decreased in CKD patients [27]. Lipid abnormalities detected in LCAT-deficient carriers mirror the ones observed in CKD patients [28], suggesting that circulating LCAT levels could predict CKD progression in severe obese individuals at early stages of renal dysfunction. Importantly, in our analyses specifically, LysoPC (18:0), LysoPC (20:3) and PC (35:3) presented the greatest predictive capacity (AUC value of one) to discriminate between OD and O patients (Figure 3).

Sphingolipids may play a role in the development and progression of CKD, and patients with CKD have higher levels of pro-oxidant and pro-inflammatory factors that could be responsible for the activation of sphingomyelinases to produce ceramides [29]. It has been reported that increased levels of plasma ceramides were associated with CKD, and ceramides may play an important role in the regulation of the inflammatory response [29]. We obtained two significantly increased ceramides, Cer (36:1) and Cer (40:1) in OD vs. O comparison, but Cer (44:1) was downregulated. The biological properties of ceramides are intensively affected by the different lengths of the acyl-chains of these free fatty acids [30]. It has been reported that ceramides with different acyl-chains may play antagonist roles [30,31] as we observed in our study.

Interestingly, among the 69 fatty acyl-derived lipids that were significant in the comparison of OD vs. O, 12 turned out to show odd-numbered fatty acyl chains (one PS, one LysoPC, four PCs, and six TGs). Odd-chain fatty acids (OCFAs) have a bacteria origin and can be endogenously synthesized from valine and isoleucine catabolism that generated propionyl-CoA, a precursor in the OCFAs biosynthesis [32]. We obtained significantly increased levels of the conditional and essential amino acids proline and isoleucine, and tyrosine, an aromatic amino acid in CKD patients with severe obesity. Elevations of branched-chain amino acids (BCAA) and aromatic amino acids, upregulated in obesity [33] and diabetes [34], have been associated with the development of metabolic diseases [35,36,37]. BCAA may be related to insulin resistance, lipids (phospholipids, fatty acids and sphingomyelins) may accumulate in the kidneys and citric acid cycle metabolites possibly indicate mitochondrial dysfunction [38]. To our knowledge, there is not much known of the association between OCFAs levels and obesity [39] or CKD, but blood levels of some specific OCFAs correlated inversely with insulin resistance and risk of diabetes [40]. Furthermore, as we mentioned, we found upregulated levels of these BCAA in OD patients, thereby the increased odd-chains lipids could reflect an impaired bacterial metabolism and increased metabolism of BCAA amino acids to produce propionyl-CoA.

BS is an effective treatment for morbid obesity, type 2 diabetes and CKD that reduces the long-term progression of CKD [9]. Bariatric, metabolic and anti- hypertensive effects are the three key pillars that underlie renoprotection in obese patients undergoing BS [10]. As previously shown in our patients, drastic weight loss induced by BS promoted a significant improvement in glucose, HDL, uric acid, C-peptide, proteinuria, and UACR parameters in OD BS vs. OD patients [12]. Furthermore, in this study BS modified the correlations presented in OD BS and OD patients. The clinical intervention promoted a change in the lipidome of CKD patients removing the existing correlation between TGs and other clinical parameters, such as cholesterol, creatinine, eGFR and proteinuria in OD BS patients. The significant decrease in the TGs levels induced by BS in OD BS patients removed the positive correlations with TGs and eGFR and proteinuria, the primary clinical parameters used for renal damage diagnosis in CKD patients with severe obesity as an indication of improvement in these patients after surgery.

With our metabolomics approach, we have revealed a decrease in serum TGs, DGs, PSs, 2 BCAA and a reduction of essential and conditional amino acids after BS. TGs, DGs and BCAA results coincide with what has been already described about the impact of BS [41,42]. Moreover, lowering circulating BCAA by means of BCAA intake reduction has been associated with a metabolic control improvement [43].

Despite the risk of complications of bariatric surgery, the use of BS for the treatment of CKD and diabetes in patients with obesity could be appropriate due to the improvement obtained in the metabolic state and the renal condition. After the surgery, we found scarce differences in TGs, DGs, PCs, PEs, PSs, Cer, and PIs (only six became significant) between OD BS and O patients (Figure 7). There are no published studies that show similarity between CKD obese patients that underwent BS compared to non-CKD obese patients. However, we revealed that some of the PCs and LysoPCs did not change in OD BS compared to OD patients (Figure 6) and therefore, LysoPC (18:0), LysoPC (20:3) and PC (35:3) could be proposed as markers of risk in CKD progression. LysoPCs have been proposed as biomarkers of nephrotoxicity in an experiment of nephrotoxicity induced by drugs in male rats [44]. These authors described that the production of oxidative species is related to the mechanism of drug-induced nephrotoxicity and the release of reactive nitrogen species (RNS) and reactive oxygen species (ROS) imbalance the oxidation and the antioxidants systems resulting in kidney damage [44]. Our results suggested that the chronic renal damage underlying metabolic alterations in obese patients were not fully reverted by the surgery. These lipid species could be associated with the non-reverted normal renal condition present at the time of measurements after surgery or they require more time for normalization. We cannot also discard an association of high levels of PCs and LysoPCs in OD BS with other unfavorable renal complications or postoperative complications that also show a direct relationship with surgical intervention [45].

In patients undergoing surgery, we could analyze first morning urine by GC-Orbitrap. First morning void is less influenced over a spot urine sample by hydration status and physical activity [46]. Moreover, the urine specimen obtained at this specific time includes an overnight fast, with the consequent reduction of the effect of the medication or the last meal [47]. In general, metabolites in urine presented only minor differences between OD BS and OD patients. We found lower levels of glutamine (Gln) and valine (Val), together with increased hippuric acid in morbidly obese subjects with CKD after BS. Urine Gln has been reported to change associated with CDK worsening condition because it was higher in 4/5 CKD vs. 2/3 CKD patients [48]. Therefore, the decrease in urine Gln and the increase of serum Gln could be associated with improved reuptake after BS. Urine Val showed the same trend as serum Val, decreased after BS. Hippuric acid, a uremic toxin, has been reported elevated in the serum of uremic patients and is associated with the progression of CKD [49], therefore, high hippuric levels found in urine suggested an improved removal of these uremic toxins after surgery.

Our results should be confirmed in a larger prospective cohort study to validate the important findings obtained in the lipidomic and the metabolomic characterization of CKD patients with severe obesity. We have identified specific lipid species, such as LysoPC (18:0), LysoPC (20:3), and PC (35:3) as possible biomarkers in CKD progression in severely obese patients, features that should be confirmed in a larger prospective study in a target analysis

Our study has several limitations, such as the small number of patients used in the study, the lack of kidney histology in most of the patients for the diagnosis of CKD, and the lack of a control group of CKD patients who did not undergo bariatric surgery to be considered as a randomized study. However, the research of the association of lipids and metabolites identified in serum and urine with the CKD amelioration obtained after a year of bariatric surgery in the same patient must be highlighted as the strength of this study.

In conclusion, in this study, the changes found in lipids and metabolites could have significant value in addition to conventional renal risk markers and may contribute to the early identification of renal malfunction in obese patients. The use of bariatric surgery in obese patients with advanced CKD might provide benefits by delaying the progression towards worse kidney decline. Even, patients who require kidney replacement therapy, BS might provide an opportunity to achieve the weight loss required to be eligible for a kidney transplant. Our results, if confirmed in larger prospective cohort studies, could eventually open the door for a lipidomic-metabolomic-based risk assessment in primary prevention of renal disease in severe obesity.

## 4. Materials and Methods

### 4.1. Study Cohort

This is an observational, prospective, single-center and not randomized study to analyze the effect produced by CKD in the metabolomic and lipidomic signature in patients with severe obesity and to evaluate the effect of weight loss in these obese patients with CKD who underwent BS (trial registration NCT02644928). CKD patients with severe obesity (OD) were selected under the consideration of the following inclusion criteria, already described by Morales et al. [12]: (i) body mass index (BMI) > 35 kg/m2 plus eGFR 30–60 mL/min and proteinuria >1 g/24 h or eGFR > 60 mL/min and proteinuria >2.5 g/24 h despite receiving maximally tolerated doses of renin-angiotensin-aldosterone system (RAAS) blocker and (ii) BMI > 40 kg/m2 with a eGFR > 30 mL/min and proteinuria >0.5 g/24 h despite receiving maximally tolerated doses of RAAS blocker. For the renal function criterion, estimated GFR (eGFR) was used (see description in Clinical Parameters test). The follow-up time was 24 months. The exclusion criteria were defined as follows: (1) Patients who had participated or were participating in another clinical trial or had taken an experimental drug in the last 28 days. (2) Patients with renal transplantation and/or chronic replacement therapy (hemodialysis and/or peritoneal dialysis). (3) Subjects with poorly controlled blood pressure (SBP > 170 mmHg or DBP > 110 mmHg). (4) Patients with a history of cardiovascular events in the past six months. (5) Patients treated with immunosuppressants. (6) Subjects with a history of renovascular disease, autoimmune diseases, cancer, drug use, or obstructive uropathy. (7) Patients who did not sign the informed consent. (8) Patients who were pregnant or lactating. (9) Patients who did not sign the informed consent; 11 patients were included in the study that fulfilled the mentioned inclusion criteria.

The impact of kidney damage in the metabolomic profile of CKD patients with severe obesity was contrasted with the incorporation of a group of 14 patients with severe obesity without CKD (O) with eligibility criteria of BMI > 35 kg/m2, eGFR > 60 mL/min and/or proteinuria < 0.30 g/24 h, did not receive any medication known to interfere with the studied variables and were matched in age with the patient group OD. Patients with severe obesity without CKD (O) did not present renal insufficiency or proteinuria.

A potential source of bias derived from the heterogeneity of CKD patients with severe obesity (OD patients) was addressed by analyzing the lipidomic total useful signal (TUS) of each CKD patient with severe obesity before the clinical intervention. The standard deviation from the lipidomic TUS was 10.90 and data were normally distributed. Further, the possible differential contribution of taking lipid-lowering drugs in the lipidomic fingerprint (473 features obtained after RSD filtration) in OD patients was analyzed and the variation obtained in the average lipidomic TUS between both CKD patients that were taking the drug and those that were not, was 4.08% for the percentage of change and the 0.06 for the Log2FC. Finally, the possible differential contribution of the method used in the bariatric surgery in the lipidome from patients who underwent Roux-en-Y gastric bypass and patients who underwent sleeve gastrectomy was analyzed. The variation in the lipidomic total useful signal between CKD patients who underwent gastric bypass or sleeve gastrectomy was 16.39% for the percentage of change and 0.26 for the Log2FC. Therefore, CKD patients with severe obesity did not present sensible differences in their lipidomic profile despite showing different comorbidities, moreover, lipid-lowering drugs and the approximation used in the bariatric surgery did not introduce a remarkable variation in these patients.

#### 4.1.1. Bariatric Surgery

After the inclusion in the study, CKD patients with severe obesity were treated with a reduced-calorie diet and those patients who did not present the expectable improvement in renal parameters were proposed for the clinical intervention. Bariatric surgery was performed in OD patients (OD BS). The specialized obesity surgery team performed two types of bariatric surgery in CKD obese subjects (OD) fitting the best surgery for the patients. Roux-en-Y gastric bypass was performed in eight CKD obese patients and sleeve gastrectomy was performed in three of these patients.

#### 4.1.2. Clinical Parameters Tests

Fasting blood samples in each patient visit were collected in the early morning to perform the following lab analysis: serum creatinine, glucose, total cholesterol, HDL, LDL (Fridewald formula), triglycerides, and uric acid. Twenty-four urine samples were collected to measure proteinuria, urea, and creatinine. First-morning urine was collected to measure the urinary albumin:creatinine ratio (UACR). Bodyweight and BMI were monitored throughout the study.

Evaluation of renal function was determined through the estimated GFR (eGFR) at all the prespecified visits (−6 and −3 months, baseline and months 3, 6, 12, and 24). eGFR was determined applying two creatinine-based equations, the Modification of Diet in Renal Disease (MDRD) and the Chronic Kidney Disease Epidemiology Collaboration (CKD-EPI) method based on a creatinine equation. Both formulas were adjusted for body surface area (BSA), a limitation for the assessment of renal function in obesity [50], and conditions associated with drastic weight change. Thus, we reversed the adjustment of the result by applying the following formula (GFR adjusted = GFR unadjusted/BSA × 1.73). BSA was calculated by the DuBois and DuBois formula (BSA = 0.007184 × Weight 0.425 × Height 0.725) [51].

A more detailed description of the body measurements and the biochemical test performed in patients were published by Morales et al. [12]. Differences between OD and O patients in the different clinical parameters presented in this work were exposed by performing a Mann-Whitney U test. Clinical parameters with a *p*-value ≤ of 0.05 were considered significant. Data distribution of clinical parameters from OD and O patients were analyzed selecting the D’Agostino & Pearson test in GraphPad Prism 8.01.

#### 4.1.3. Study Design

The present study has been developed with serum and urine samples of CKD patients with severe obesity before bariatric surgery (OD patients) and the same patients with CKD after bariatric surgery (OD BS patients), and patients with severe obesity without CDK (O patients). Eleven patients with CKD and 14 patients without CKD were included in the study. Serum samples and urine samples from OD patients were collected before the bariatric surgery (baseline). Serum samples and urine from OD BS patients were collected after the clinical intervention. Serum samples were collected from O patients. First morning urine was collected from eight patients with CKD before and after the surgery. For O patients only 24-h urine was available. Therefore, only urine from CKD patients before and after surgery was included in the analysis as it has been reported that the use of different urine specimens can result in variability in the urine metabolome [47].

In the present work, samples obtained from CKD patients with severe obesity before bariatric surgery will be referred to as OD (obese disease). Samples obtained from CKD patients with severe obesity after bariatric surgery will be referred to as OD BS (obese disease bariatric surgery). Samples obtained from non-CKD patients with severe obesity will be referred to as O (obese).

#### 4.1.4. Samples Collection

Blood samples were collected in the early morning in serum tubes provided with a gel serum separator. Then, samples were centrifuged (3500 rpm, 15 min at 4 °C), aliquoted and stored at −80 °C until extraction. Urine, first void in the morning, was collected, aliquoted and stored at −80 °C until extraction. Sample collection procedures were in accordance with the Helsinki declaration and approved by the local ethics committee.

### 4.2. Lipidomic Untargeted Analysis in Serum Samples by Liquid Chromatography Coupled to Mass Spectrometry (LC-MS)

#### 4.2.1. Lipidomic Extraction, Sample Preparation

A specific method developed to analyze the lipidome was used with the serum samples [52]. The lipidomic fraction was extracted following a modified Folch technique [53]; 10 µL of each sample were added to 10 µL of 0.9% NaCl and 120 µL of CHCl3: MeOH (2:1, *v/v*) containing a 2.5 ppm solution of different lipids standards (to improve precision of quantitative analysis, data normalization and control instrument). The solution contained the following standards purchased from Sigma Aldrich: LPC(17:0) (1-heptadecanoyl-2-hydroxy-sn-glycero-3-phosphocholine), PC(17:0/17:0) (1,2-diheptadecanoyl-sn-glycero-3-phosphocholine), PE(17:0/17:0) (1,2-diheptadecanoyl-sn-glycero-3-phosphoethanolamine), Cer(d18:1/17:0) (N-heptadecanoyl-D-erythrosphingosine), PC(16:0/d31/18:1) (1-palmitoyl-d31-2-oleoyl-sn-glycero-3-phosphocholine), SM(d18:1/17:0) (N-heptadecanoyl-D-erythro-sphingosylphosphorylcholine), CE(17:0) (Cholest-5-en-3b-yl, heptadecanoate). Additionally, TG (17:0/17:0/17:0) 1,2,3-triheptadecanoyl-sn-glycerol, Triheptadecanoin) were acquired from Larodan (Solna, Sweden). Samples were randomized, vortex-mixed and put on ice for 30 min. After that, samples were centrifuged (9400× *g*, 3 min, 4 °C). Then, 60 µL of the lower layer of each sample was transferred to a glass vial with an insert and 60 µL of CHCl3: MeOH (2:1, *v*/*v*) was added. Samples were stored at −80 °C until analysis.

Equipment robustness and lipid quantification were evaluated with calibration curves of 7 points (Concentrations of 0.1, 0.5, 1, 2, 3, 4, 5 ppm) prepared with LPC(18:0) (1-octadecanoyl-sn-glycero-3-phosphocholine), Cer (d18:1/18:1 9Z)) (N-Oleoyl-D-sphingosine), PC (18:0/18:0) (1,2-Distearoyl-sn-glycero-3-phosphocholine), PE (16:0/16:0) (1,2-Dipalmitoyl-sn-glycero-3-phosphocholine), LysoPE (18:1(11Z)) (1-O-Palmityl-sn-glycero-3-phosphocholine), CE (16:0) (Cholest-5-en-3β-yl hexadecanoate), CE (18:0) (Cholest-5-en-3β-yl octadecanoate), CE (18:1) (Cholest-5-en-3b-yl octadecenoate), CE (18:2) (Cholest-5-en-3β-yl octadecadienoate), TG (16:0/16:0/16:0) (1,2,3-trihexadecanoyl-sn-glycerol), TG (18:0/18:0/18:0) (1,2,3-trioctadecanoyl-sn-glycerol) purchased from Sigma Aldrich and PC (16:0/16:0) acquired from Larodan (Solna, Sweden).

The stability and reproducibility of the system were checked with quality control (QC) samples prepared with patient samples. Quality control samples were extracted in the same way as ordinary samples. QC samples were prepared by combining the leftover of each sample after centrifugation. The total was centrifuged, 60 µL of the lower layer was transferred to the vials with insert, and 60 µL of CHCl3: MeOH (2:1, *v/v*) was added. Samples were stored at −80 °C until analysis. To ensure that data reflects the biological complexity of the samples, consequent signals derived from the extraction and the instrumental analysis were assessed with extraction blanks prepared as patient samples without any biological trace.

#### 4.2.2. UHPLC-ESI-Q-TOF-MS Analysis

An ultra-high-performance liquid chromatography-electrospray ionization quadrupole time-of-flight (UHPLC-ESI-Q-TOF-MS) was used to analyze the samples on positive ionization mode based on previously set conditions [52]. Samples were analyzed by duplicate. UHPLC system was an Agilent Infinity 1290 provided by Agilent Technologies (Santa Clara, CA, USA) equipped with a multisampler (kept at −10 °C). Needle wash solutions were performed with 10% DCM in MeOH and ACN:MeOH:IPA:H_2_O (1:1:1:1, *v*/*v*/*v*/*v*), and 0.1% of HCOOH after each injection for 7.5 s. The system was equipped with a column thermostat (maintained at 50 °C) and a quaternary solvent manager. An ACQUITY UPLC BEH C18 column was used for separations (2.1 mm × 100 mm, particle size 1.7 μm) purchased at Waters (Milford, CT, USA). Injection volume was 1 μL and the flow rate was established at 0.4 mL/min. Mobile phases were composed of (A) H_2_O + NH_4_AC 10 mM + 0.1% HCOOH and (B) ACN: IPA (1:1, *v*/*v*) + NH_4_AC 10 mM + 0.1% HCOOH. The gradient was from 0 to 2 min 35–80% B, 2 min to 7 min 80–100% B and 7 to 14 min 100% B. A re-equilibration of 7 min was performed after each run to bring the system to initial conditions (35% B). Mass Spectrometer was an Agilent 6545 quadrupole time-of-flight (Q-TOF) mounted with a dual jet stream electrospray (dual ESI) ion source interface. Nitrogen was obtained from a nitrogen generator (PEAK Scientific, Renfrewshire, Scotland, UK) as a sheath gas at a flow rate of 11 l/min at 379 °C. As a Collision gas was used Pure Nitrogen (6.0) from Praxair (Fredericia, Denmark). The capillary voltage was maintained at 3600 V and nozzle voltage was kept at 1500 V. Reference mass solution was prepared in consonance with Agilent guidelines, including ions at *m*/*z* 121.0509 and 922.0098. The second nebulizer was used to introduce the solution in the dual ESI ion source through the isocratic pump at a constant flow rate of 4 mL/min (split to 1:100 before nebulization). The acquisition mass range was 100 to 3000 *m*/*z*. The instrument used the extended dynamic range with an estimated resolution of 30.000 FWHM measured at 1521.9715 *m*/*z* (included in tune mixture) when instrument calibration was performed. Extracted blanks and QCs samples were disposed of throughout the instrumental analysis. Calibration curves were measured at the end of the analysis. Data acquisition was performed with Agilent MassHunter B.08.00.

#### 4.2.3. MS Signals Processing

MS Signals were processed with MZ Mine 2 (version 2.54) [54] as previously described for LC-MS analysis [52]. Mass detection was performed with a noise level set at 6 × 10^2^. After that, chromatogram builder was done setting min-height at 6 × 10^2^ and *m*/*z* tolerance at 0.007 *m*/*z* or 7.0 ppm. Before doing the deconvolution, internal standards were checked in some random samples to evaluate their stability and signal. Chromatogram deconvolution was done with the local minimum search algorithm with the following settings: chromatogram threshold at 70%, search minimum in RT range (min) 0.03, minimum relative height 1.0%, minimum absolute height 6 × 10^2^, min ratio of peak top/edge 1.21 and peak duration range (min) was established from 0.00 to 2.00. Isotopes were performed under these conditions: *m*/*z* tolerance 0.007 *m*/*z* or 7.0 ppm, retention time tolerance 0.07 absolute (min) and a maximum charge of 2. The Hierarchical Alignment (GC) was selected to do the alignment with these values: *m*/*z* tolerance 0.009 *m*/*z* or 9.0 ppm and the weight for RT was set at 1. Peak list row filter was the option selected for the filtering with these parameters: *m*/*z* from 300 to 1200 and retention time from 2.00 to 12.00. Gap filling was performed with an Intensity tolerance of 50.0%, an *m*/*z* tolerance of 0.01 *m*/*z* or 12.0 ppm and a retention time tolerance of 0.15 absolute (min). Features with low signal (below 1 × 10^3^), repeated and that were not present at least in more than 80% of samples were not included in the analysis. No missing values were detected in the final list of features selected. Lipid identification was done using an in-house library through the custom database search option (147 compounds were identified of 604 features) with an *m*/*z* tolerance set at 0.05 *m*/*z* or 20 ppm and a retention time tolerance established at 0.10 absolute (min). Not mentioned parameters in each point of the process were set with default values.

#### 4.2.4. Data Pre-Treatment

Pre-treatment of data. Relative standard deviation (RSD) was calculated for QC samples. Features that presented an RSD of less than 30% were selected (473 lipid metabolites were filtered out of 604 features). Normalization was performed in a different way depending on if the feature had been previously annotated through the in-house library or was an unknown compound. Annotated compounds were normalized against the same subclass of the IS included in the sample preparation. LPC were normalized against LPC (17:0), PC were normalized against PC (17:0/17:0), PE were normalized against PE (17:0/17:0), Cer were normalized against Cer (d18:1/17:0), SM were normalized against SM (d18:1/17:0), CE were normalized against CE (17:0) and TG were normalized against TG (17:0/17:0/17:0). Unknown features eluted from 2 to 5.5 min were normalized against LPC (17:0), from 5.5 to 7.5 min were normalized against PC (17:0/17:0) and from 7.5 to 12 min were normalized against TG (17:0/17:0/17:0). Average was performed in duplicate data from each sample in all the features obtained to maintain a central tendency of MS data.

#### 4.2.5. Statistical Analysis

Univariate (UVA) and multivariate statistical analysis (MVA) were performed to expose the difference between groups after filtration with RSD, normalization and averaged calculation of MS Data, as previously described by Gonzalez-Riano et al. [55].

All multivariate analyses (MVA) were performed with SIMCA-P v16.0 (Sartorius-Umetrics, Umeå, Sweden). Unsupervised (principal component analysis, PCA) and supervised (orthogonal projection on latent structures/partial least squares discriminant analysis, OPLS-DA) MVA were carried out. For PCA, the dataset was unit-variance (UV) scaled. PCA was performed to assess the spontaneous clustering of patient samples and QC samples using the complete dataset of variables of all samples. After careful evaluation of the quality of the model and the clustering of the QCs, supervised models were built. For maximizing the classification power of metabolites, OPLS-DA models were generated between OD vs. O, OD BS vs. OD, and OD BS vs. O (data not shown). Before building the models, UV scaling was applied to all datasets. The quality of the OPLS-DA models was evaluated by the values of Q2 (estimation of the predictive ability of the model), and R2 (the explained variance). Validation of all the models was performed by means of cross-validation and *p*-value, both obtained from the CV-ANOVA tool. The variables’ influence on projection (VIPs) were obtained for each variable from the OPLS-DA models built in the OD vs. O and OD BS vs. OD comparisons.

Univariate analysis (UVA) was performed applying different statistical tests depending on the comparison evaluated. For OD vs. O and OD BS vs. O a non-parametric Mann–Whitney U test was used. For OD BS patients vs. OD patients, a Wilcoxon non-parametric paired test was applied. The Benjamini–Hochberg correction was applied to the *p*-value to control the false discovery rate (FDR). The tests were applied to all the individual features using in-house scripts for MATLAB R1080a (MathWorks, Natick MA, USA).

Monofactorial and multifactorial tests were then employed to select the compounds with the highest biological relevance, and the features were considered relevant when either *p*-value ≤ 0.05 in UVA or VIP value > 1 in MVA were seen. In addition, the Jackknife confidence interval (no zero-value inclusion) was considered to interpret the confidence of the clustering agreement measurements obtained on the OPLS-DA models.

#### 4.2.6. Annotation of Unknown Features

Unknown features were annotated by LC-MS/MS in positive and negative ionization using QC samples. The acquisition mass range was set from 50 to 3000 *m*/*z* in both ionizations. The capillary voltage was maintained at 3600 V in positive and 5500 V in negative. In positive mode, 1 µL of QC sample was injected per method and 5 µL was injected in negative mode. Features were annotated in negative ionization using the following adducts per lipid subclass: LPC/PC and SM with [M+FA-H]^−^, PI with M-H and M+FA-H, PG with M-H and M+Cl, PS with M-H and M-H_2_O-H, Cer with M-H, M+HCOO^−^, M+FA-H and M+Cl. Diverse spectral libraries were used to annotate the unknown features, such as MetFrag [56], MassBank (https://massbank.eu/MassBank/, accessed on 10 November 2021), PubChem (https://pubchem.ncbi.nlm.nih.gov/, accessed on 10 November 2021), HMDB [57] and bibliographical resources [58]. Furthermore, DG and TG were putative annotated studying the adduct formation of these lipid species [59].

### 4.3. Polar Metabolites Untargeted Analysis in Serum and Urine Samples by Gas Chromatography Coupled to High-Resolution Accurate Mass Spectrometry (GC-HRAM-MS)

#### 4.3.1. Extraction of Polar Metabolites, Sample Preparation

Serum and urine samples were extracted following the same method published by Castillo et al. [60] with minor modifications respecting the matrix used; 30 µL of serum (150 µL of urine) of each sample were randomized and vortex-mixed with 400 µL of MeOH at −20 °C containing 1 ppm of the following internal standards: heptadecanoic acid, valine-d8, succinic acid-d4, and glutamic acid-d5 (Sigma-Aldrich, Saint Louis, MO, USA). Samples were incubated on ice for 30 min and centrifuged (9600 rpm, 3 min). After that, 350 µL (400 µL for urine) of the supernatant of each serum sample were transferred to a V-shaped GC-vial. The stability and reproducibility of the system were checked with QC samples prepared to collect from all the extracts the same quantity of the remaining supernatant. Afterward, QC samples were vortex-mixed, centrifuged and 350 µL (400 µL for urine) of the supernatant of each aliquot were transferred to a V-shaped GC-vial. To ensure that data reflects the biological complexity of the samples, consequent signals derived from the extraction and the instrumental analysis were assessed with extraction blanks prepared as patient samples without any biological trace.

Derivatization. Supernatants were evaporated to dryness in a nitrogen flow. Then, samples were converted to trimethylsilyl (TMS) and methoxime (MEOX) derivate(s). Consequently, 25 µL of MOX reagent in pyridine (20 mg/mL) were added, samples were vortex-mixed and incubated for 60 min at 45 °C. After oximation, silylation was performed adding 25 µL of MSTFA, samples were vortex-mixed and incubated for 60 min at 45 °C. To quantify the relative elution times of the different features in gas chromatography, 25 µL of a mixture containing odd n-alkanes from C11 to C25 (10 ppm in hexane) was added to the vials before injection together with the injection standard 4,4′-Dibromooctafluorobiphenyl (DBOFB, Sigma-Aldrich, Saint Louis, MO, USA).

#### 4.3.2. GC-HRAM-MS Analysis

A gas chromatography-high-resolution/accurate-mass spectrometry (GC-HRAM-MS) coupled equipment was used to analyze the patient samples (serum and urine) based on previously set conditions adapted to the idiosyncrasy of our samples [61]. GC-HRAM-MS analyses were performed in a Q Exactive GC Orbitrap system (Thermo Scientific Waltham, MA, USA), mounted with a Rxi Guard column purchased at Restek (10 m × 0.37 mm, 0.25 µm i.d.) and a capillary column provided by Agilent Technologies (30 m, 0.25 mm, 0.25 µm i.d.). Injection (1µ) was done in splitless mode with a TriPlus RSH autosampler system provided by Thermo. The oven temperature was kept at 70 °C for the first 5 min. Then, the temperature was increased to 260 °C (10 °C/min) to reach in the final step 300 °C (40 °C/min) for 5 min. The carrier gas used was Helium with a flow of 2.0 mL/min. Scan range and resolution were adjusted to 50–500 *m*/*z* and 60,000 respectively. MS Detector was operated in EI positive mode. The ion source and the transfer line were kept at 280 °C. QC samples were injected after every seven samples in serum analysis and after three in urine analysis. Suitability blank (hexane) and process blank (extraction blank) were analyzed at the beginning and the end of the analysis.

#### 4.3.3. MS Signals Processing for Serum Samples

Data pre-processing was done with MZ Mine (version 2.54) adapting the ADAP method to the peculiarity of these samples [62]. Mass detection was performed with a noise level set at 5 × 10^4^. Then, ADAP chromatogram builder was done setting the min group size in # of scans at 10, group intensity threshold was set at 1.5 × 10^7^, min highest intensity was established at 1.5 × 10^7^ and *m*/*z* tolerance was adjusted at 0.01 *m*/*z*. The next step was to do smoothing with a filter width of 25. Chromatogram deconvolution was performed with the following parameters: *m*/*z* center calculation Median, Algorithm Wavelets (ADAP), S/N threshold at 10, min feature height at 1.5 × 10^6^, coefficient/area threshold at 800, peak duration range from 0.01 to 0.40 and RT wavelet range from 0.00 to 0.06. After that, spectral deconvolution was done under the hierarchical clustering option with the following criteria: Min cluster distance (min) at 0.01, min cluster size at 2, min cluster intensity at 1000, find shared peaks option was ticked, min edge-to-height ratio at 0.3, min delta-to-height ratio at 0.2, min sharpness at 10, shape-similarity tolerance at 70 and *m*/*z* ranges from 73.0 to 73.99 and 147.0 to 147.99 were excluded. Alignment was done under ADAP aligner (GC) option parameters were adjusted as follows: Min confidence at 0.3, retention time tolerance at 0.2 (absolute min), *m*/*z* tolerance was set at 0.003 *m*/*z*, score threshold at 0.7 and score weight at 0.1. Next to the alignment, gap filling was performed with the peak finder (multithreaded) option selected: intensity tolerance at 2.0% and *m*/*z* tolerance was set at 0.005 *m*/*z* and 0.2 ppm. Features with low signal (below 1 × 10^3^), that repeated and that were not present at least in more than 80% of samples were not included in the analysis. No missing values were detected in the final list of features selected. Polar metabolites identification was done using an in-house library through the custom database search option (27 compounds were annotated of 197 features) with an *m*/*z* tolerance set at 0.05 *m*/*z* or 30 ppm and a retention time tolerance established at 0.30 absolute (min). Not mentioned parameters in each point of the process were set with default values.

#### 4.3.4. MS Signals Processing for Urine Samples

MS signals were processed with MZ Mine (version 2.54) repeating the same steps followed in serum samples with the ADAP method. Initially, crop filtering was set from min 9.5 to min 22 to work with signals contained just in that range of retention time (RT). Mass detection was performed with a noise level set at 1 × 10^4^. Then, ADAP chromatogram builder was done setting the min group size in # of scans at 10, group intensity threshold was set at 1 × 10^5^, min highest intensity was established at 1 × 10^5^ and *m*/*z* tolerance was adjusted at 0.01 *m*/*z* or 0.0 ppm. The next step was to do smoothing with a filter width of 25. Chromatogram deconvolution was performed with the following parameters: *m*/*z* center calculation Median, Algorithm Wavelets (ADAP), S/N threshold at 10, min feature height at 1 × 10^4^, coefficient/area threshold at 800, peak duration range from 0.01 to 0.40 and RT wavelet range from 0.00 to 0.06. Spectral deconvolution, alignment and gap-filling were performed with the same parameters used in serum samples. Features with low signal (below 1 × 10^3^), repeated and that were not present at least in more than 80% of samples were not included in the analysis; 94 features were obtained after signals’ processing. Two missing values were detected in the final list of features selected and were treated with the k-nearest neighbors algorithm (KNN) using an in-house script for MATLAB. Identification was done with the same in-house library used with serum samples, but no hits were found with urine samples. Not mentioned parameters in each point of the process were set with default values.

#### 4.3.5. Data Pre-Treatment

Serum samples: MS data were normalized against valine-d8, data was strengthened after normalization improving RSD and decreasing data variation. Features were filtered with RSD calculated for QC samples. Features that presented an RSD of less than 40% were selected (113 polar metabolites were filtered out of 197 features). Urine samples: Probabilistic quotient normalization method (PQN) [63] was used to normalize MS Data. PQN was applied using an in-house MATLAB script considering all the variables of the profiles. Features were filtered with RSD calculated for QC samples. Features that presented an RSD of less than 40% were selected (48 polar metabolites were filtered out of 94 features).

#### 4.3.6. Statistical Analysis

Statistical Analyses in serum and urine analyzed by GC-HRAM-MS were performed applying the same principles described in LC-MS analysis with certain variations (see Section 4.2.5).

All multivariate analyses were performed with SIMCA-P v16.0. For PCA, the data set was unit-variance (UV) scaled. After the careful evaluation of the PCA models, OPLS-DA models were generated between OD vs. O, OD BS vs. OD, and OD BS vs. O (data not shown) in serum and OD BS vs. OD in urine. For the serum analyses, logarithmic transformation and Pareto scaling were applied to the OD vs. O dataset, and UV scaling was applied to the OD BS vs. OD dataset. For the urine analyses (OD BS vs. OD), logarithmic transformation and centroid scaling were applied. Validation of all models was performed with the CV-ANOVA tool provided by the software. Validation of all the models was performed by means of cross-validation and *p*-value, both obtained from the CV-ANOVA tool. The variables’ influence on projection (VIPs) were obtained for each variable from the OPLS-DA models.

Univariate analysis (UVA) was performed applying different statistical tests depending on the comparison evaluated. For OD vs. O and OD BS vs. O, a non-parametric Mann–Whitney U test was used. For OD BS patients vs. OD patients, a Wilcoxon non-parametric paired test was applied. The tests were applied to all the individual features using in-house scripts for MATLAB R1080a (MathWorks, Natick, MA, USA).

Due to the dispersion found between groups comparisons and the small differences between groups, the selection criteria for significant compounds were less restrictive than the one used in LC-MS analysis. The Benjamini–Hochberg correction was not applied, and features were considered biologically relevant when either *p*-value ≤ 0.05 in UVA or VIP value > 1 in MVA were seen. Therefore, monofactorial and multifactorial tests were considered to select the compounds with the highest biological relevance.

#### 4.3.7. Annotation of Unknown Features

Features were putatively annotated matching their spectra with the spectra available in the following databases: GOLM Metabolome Database [64], default parameters were used with a VAR5 column type and the calculated Kovats retention index (RI) for the compounds was entered in the database to improve the annotation. MS Search (2.3 version by NIST Mass Spectrometry Data Center) was used with HMBD [57], Fiehn (RTX5 Fiehn Library for Metabolic Profiling) and NIST (14 version) libraries. RI was also considered. Mass Bank through peak list option was consulted to improve the annotation.

### 4.4. Correlation, Curve ROC Test Analyses and Heatmaps

Correlation analysis was performed with normalized peak intensity values from significant features obtained after univariate and multivariate statistical analyses in all comparisons in serum LC-MS and GC-HRAM-MS analysis and urine GC-HRAM-MS analysis. Normalized peak intensity values were analyzed against biochemical parameters of OD, OD BS and O patients applying a Pearson Correlation test per group of patients using an in-house script for R (version 4.0.3). Correlations with a *p*-value ≤ of 0.05 were selected as significant.

ROC analysis was performed with normalized peak intensity values from significant features obtained after UVA and MVA analyses in OD vs. O and OD BS vs. OD comparisons in serum and urine LC-MS and GC-HRAM-MS analyses. ROC analysis was built in Metaboanalyst 5.0 [65] under the “Classical univariate ROC curve analyses” option with default settings. Features with an AUC value ≥ of 0.90 in features obtained in LC-MS analysis and ≥ 0.80 in those obtained in GC-HRAM-MS analysis, were selected as representative for their high discriminatory power classifying patients before and after BS.

Heatmaps were created with normalized peak intensity values from significant features obtained after UVA and MVA analyses in OD vs. O and OD BS vs. OD comparisons in serum LC-MS and GC-HRAM-MS analyses. Metaboanalyst 5.0 was used to generate the different heatmaps [65]. The software was set with Euclidean distance; Ward clustering method and samples were reorganized. Not mentioned parameters in each point of the process were set with default values.

### 4.5. Lipidomic and Amino Acid Pathways 

The lipidomic pathways (Figures S1 and S3) are a modified version of the “Glycerolipids and Glycerophospholipids” scheme from Wikipathways [66]. The amino acid pathways (Figures S2 and S4) were created taking “Amino Acid Biosynthesis” pathway from Biochemistry as a reference [67].

## Figures and Tables

**Table 1:** Body weight and biochemical measurements in OD, OD BS and O patients. Data are presented as mean ± standard deviation, median or percentages.

	Group		
Parameters	Patients without CKD	CKD Patients	
		CKD Patients before BS	CKD Patients after BS
Acronym	O	OD	OD BS
n	14	11	
Age (years), mean ± SD (range)	51.76 ± 10.92 (35–66)	53.09 ± 15.16 (29–71)	54.09 ± 15.16
Gender (Male/Female) (%)	38.47/61.53	66.64/33.36	66.64/33.36
Body weight (kg), mean ± SD (range)	120.51 ± 16.96 (84–152)	116.72 ± 25.33 (93.5–170)	81.07 ± 22.42 (65–125) #
BMI (kg/m2), mean ± SD (range)	42.9 ± 3.72 (36.48–50.0)	41.9 ± 5.98 (36.6–53.3)	28.6 ± 5.69 (22.68–39.78) #
Diabetes mellitus (%)	28.6	63.6	9.1
Hypertension (%)	35.7	90.9	63.6
Lipid-lowering drugs (%)	14.3	63.6	18.1
Glucose (mg/dL), median (range)	100 (79–171)	174 (98–299) *	86 (67–141) #
HbA1c (%), mean ± SD	6.02 ± 0.72	7.46 ± 1.81 *	5.55 ± 0.83 #
Cholesterol (mg/dL), mean ± SD	183 ± 34.74	194 ± 49.62	160 ± 41.70 #
HDL (mg/dL), median (range)	45.9 (35.0–94.0)	35 (21.6–57.3) *	45 (27–78) #
LDL (mg/dL), mean ± SD	103.63 ± 26.19	98.50 ± 37.70	89.27 ± 37.85
TG (mg/dL), mean ± SD	183.46 ± 126.29	314.54 ± 106.16 *	116.36 ± 45.82 #
Uric acid (mg/dL), mean ± SD	5.52 ± 0.83	7.10 ± 1.69 *	5.64 ± 1.10 #
Serum Creatinine (mg/dL), mean ± SD	0.80 ± 0.18	1.16 ± 0.43 *	1.03 ± 0.39
eGFR (mL/min), mean ± SD	94.18 ± 20.35	73.02 ± 30.76 *	80.72 ± 31.02
Proteinuria (g/24 h), median (range)	0.14 (0.10–0.53)	1.48 (0.77–11.40) *	0.68 (0.34–3.78) #
UACR (mg/g), median (range)	7.6 (3.6–110.4)	1004 (158.0–6825) *	321.79 (38.69–3104) #

Data distribution: Normally distributed data are presented as means. Data that were not normally distributed are presented as medians (Glucose, HDL, Proteinuria and UACR). Abbreviations: LDL, low density lipoprotein; HDL, high density lipoprotein; TG, Triglycerides; eGFR, estimated glomerular filtration rate. UACR: urinary albumin:creatinine ratio. Clinical parameters were considered significant when at least reach a *p* value ≤ of 0.05. Significant symbols: asterisk (*), significant between OD vs. O patients. Hash (#), significant between OD BS vs. OD patients.

**Table 2:** Correlation analyses summarize the table for serum LC-MS in OD vs. O comparison. The direction of the relationship was assessed considering the general trend, through a positive or negative correlation, of the significant correlations obtained for each metabolite for each clinical parameter. Complete correlation data for each significant feature obtained for each group of patients against the different clinical parameters can be found in Table S3.

	O		OD	
	Highlighted	Relationship	Highlighted	Relationship
**Glucose**	Cer, PE, LysoPC, DG, TG	Positive	—	—
**Cholesterol**	Cer, PS, PC	Positive	TG	Positive
**LDL**	—	—	LysoPC	Positive
**Uric Acid**	LysoPC, PC	Positive	TG	Positive
**Creatinine**	SM, PC, LysoPC, TG	Negative SM, Positive PC, LysoPC, TG	TG	Positive
**eGFR**	—	—	TG	Negative
**Proteinuria**	—	—	DG, TG	Positive
**UACR**	SM, PC	Positive	—	—

(—) Lack of significant correlation. Abbreviations: TG, Triglycerides. DG, Diglycerides. PC, Phosphatidylcholine. LPC, Lysophosphatidylcholine. Cer, Ceramide. PE, Phosphatidylethanolamine. PS, Phosphatidylserine. SM, Sphingomyelin. UACR: urinary albumin:creatinine ratio.

**Table 3:** Correlation analyses summarize table for serum LC-MS in OD BS vs. OD comparison. Direction of the relationship was assessed considering the general trend, through a positive or negative correlation, of the significant correlations obtained for each metabolite for each clinical parameter. Complete correlation data for each significant feature obtained for each group of patients against the different clinical parameters can be found in Table S3.

	OD BS		OD	
	Highlighted	Relationship	Highlighted	Relationship
**Glucose**	—	—	—	—
**Cholesterol**	SM, LysoPC, PI	Positive	TG	Positive
**LDL**	SM	Positive	LysoPC	Positive
**Uric Acid**	DG, TG (medium)	Positive	TG (short)	Positive
**Creatinine**	—	—	TG	Positive
**eGFR**	—	—	TG	Negative
**Proteinuria**	—	—	DG, TG	Positive
**UACR**	—	—	—	—

(—) Lack of significant correlation. Abbreviations: TG, Triglycerides. DG, Diglycerides. LPC, Lysophosphatidylcholine. SM, Sphingomyelin. PI, Phosphatidylinositol.

## Data Availability

The data supporting the findings of this study are openly available in Metabolomics Workbench [68] repository at http://www.metabolomicsworkbench.org/ (accessed on 10 November 2021) with the following access number for the studies: Serum LC-MS: ST001824, Serum GC-HRAM-MS: ST001825 and Urine GC-HRAM-MS: ST001826.

## Supplementary Materials

The following are available online at https://www.mdpi.com/article/10.3390/metabo11120836/s1. Table S1: ROC Curve test for OD vs. O comparison, Table S2: ROC Curve test for OD BS vs. O D comparison, Table S3: Complete correlation analysis results for OD, OD BS and O patients compared with clinical parameters highly related to kidney dysfunction, Table S4: Statistically significant metabolites found in UVA and MVA statistical performed in OD vs. O and OD BS vs. OD comparisons. Figure S1: lipidomic pathway in serum analyzed by LC-MS in OD vs. O, Figure S2: amino acid pathway in serum analyzed by GC-HRAM-MS in OD vs. O, Figure S3: lipidomic pathway in serum analyzed by LC-MS in OD BS vs. OD, Figure S4: amino acid pathway in serum analyzed by GC-HRAM-MS in OD BS vs. OD, Figure S5: Unsupervised PCA model, Figures S6–S10: Supervised OPLS-DA models.
