# Supplementary material for: Lipidomic and Metabolomic Signature of Progression of Chronic Kidney Disease in Patients with Severe Obesity
Source: Metabolites. 2021 Dec 3;11(12):836. doi: 10.3390/metabo11120836 (PMC8707539; doi:10.3390/metabo11120836)
Supplement: Supplementary file 1 [file metabolites-11-00836-s001.zip › metabolites-1483861-supplementary.pdf]

Supplementary Information

Lipidomic and metabolomic signature of progression of Chronic Kidney Disease in patients with severe obesity

Authors:

Borja Lanzon [1], Marina Martin-Taboada [1], Victor Castro-Alves [2], Rocio Vila-Bedmar [1], Ignacio González de Pablos [3], Daniel Duberg [2], Pilar Gomez [4], Elias Rodriguez [4], Matej Orešič [5,6], Tuulia Hyötyläinen [2], Enrique Morales [3,7,8], Francisco J. Ruperez [9] and Gema Medina-Gomez [1,10,*]

[1] LIPOBETA Group, Department Basic Sciences of Health, Faculty of Sciences of Health, Universidad Rey Juan Carlos, 28922 Alcorcón, Madrid, Spain; borja.lanzon@urjc.es (B.L.); marina.martin@urjc.es (M.M.-T.); rocio.vila@urjc.es (R.V.-B.)

[2] School of Science and Technology, Örebro University, 702 81 Örebro, Sweden; Victor.Castro-Alves@oru.se (V.C.-A.); Daniel.Duberg@oru.se (D.D.); Tuulia.Hyotylainen@oru.se (T.H.)

[3] Department of Nephrology, University Hospital 12 de Octubre, 28041 Madrid, Spain; igp.snurse@gmail.com (I.G.d.P.); emorales@salud.madrid.org (E.M.)

[4] Department of Surgery, Hospital Universitario 12 de Octubre, 28041 Madrid, Spain; pilgom@hotmail.com (P.G.); elias.rodriguezcue@salud.madrid.org (E.R.)

[5] School of Medical Sciences, Örebro University, 702 81 Örebro, Sweden; Matej.Oresic@oru.se

[6] Turku Bioscience, University of Turku and Åbo Akademi University, 20520 Turku, Finland

[7] Research Institute of University Hospital 12 de Octubre (imas12), 28041 Madrid, Spain

[8] Department of Medicine, Complutense University of Madrid, Madrid, Spain

[9] Centro de Metabolómica y Bioanálisis (CEMBIO), Universidad San Pablo-CEU, CEU Universities, 28668 Boadilla del Monte, Spain; ruperez@ceu.es

[10] LAFEMEX Laboratory, Área de Bioquímica y Biología Molecular, Departamento de Ciencias Básicas de la Salud, Facultad de Ciencias de la Salud, Universidad Rey Juan Carlos, 28922 Alcorcón, Spain

* Correspondence: gema.medina@urjc.es; Tel.: +34-91-488-8632

Table of content

ROC Curve test: significant features obtained in OD vs. O and OD BS vs OD patients comparisons in LC-MS and GC-HRAM-MS analyses for serum and urine were evaluated in a ROC Curve test performed with Metaboanalyst 4.0. **Table S1** and **Table S2** showed ROC Curve test results for OD vs. O and OD BS vs OD patient comparisons, respectively.

Lipidomic and amino acid pathways: **Figure S1** and **Figure S2**, lipidomic and amino acid pathways for discriminating features found in serum in OD vs. O comparison. **Figure S3** and **Figure S4**, lipidomic and amino acid pathways for discriminating features found in serum in OD BS vs. OD comparison.

Correlation analysis: detailed significant correlations obtained for OD, OD BS and O patients compared with clinical parameters highly related to kidney dysfunction. **Table S3**, correlation analyses in serum LC-MS for OD, OD BS and O patients.

Figure S5. Unsupervised PCA model: features of OD, OD BS and O patients obtained in serum for LC-MS analysis were analyzed in a multivariate statistical analysis (MVA) performed in SIMCA-P 16.0. White triangles, O patients; black circles, OD patients; soft grey squares, OD BS patients. Analyzing all the lipids simultaneously obtained after RSD filtration through a PCA model, the lipidomic profiles between obese patients with or without CKD were different. The samples corresponding to OD and OD BS patients were perfectly separated in the ellipse by the primary variable (vertical) and the second variable (horizontal). Bariatric surgery in patients with CKD induced changes in their lipidome that allowed these patients to approach the position of obese patients without kidney disease (O) in the ellipse. These changes in the individuals after bariatric surgery practically eliminated the variable that allowed them to separate vertically from obese patients without kidney disease before the intervention.

Supervised OPLS-DA models: Figures S6 to S10. Supervised OPLS-DA models were performed with SIMCA-P 16.0: variables obtained after RSD filtration in serum and urine were analyzed in a multivariate statistical analysis (MVA). OPLS-DA models were yield in OD vs. O and OD BS vs. OD comparisons in the serum analyzed by LC-MS and GC-HRAM, and the urine analyzed by GC-HRAM-MS.

Table S4. Statistically significant metabolites found in UVA and MVA statistical analysis performed in OD vs. O and OD BS vs. OD comparisons.

Author attributions. Graphical abstract has been designed with resources from Flaticon.com. Patient with obesity, urine sample and human figure were created by Freepink. Blood drop was created by Pixel Perfect.

Table S1. Curve ROC analysis. Analysis built with significant features obtained in OD vs. O comparison in serum LC-MS and GC-HRAM-MS analyses. Features were selected with AUC values above 0.9 for LC-MS and 0.8 in GC-HRAM-MS. Abbreviation: TG, Triglycerides. PC, Phosphatidylcholine. LPC, Lysophosphatidylcholine. PE, Phosphatidylethanolamine. PS, Phosphatidylserine. PI, Phosphatidylinositol. SM, Sphingomyelin.

Serum LC-MS

**Feature**	**AUC**	**T-tests**	**Log2 FC**
LysoPC (18:0)	1	1.50E-06	-1.37
LysoPC (20:3)	1	4.03E-05	-1.52
PC (35:3) / (18:2/17:1)	1	1.77E-06	-0.52
LysoPC (14:0)	0.99	1.35E-05	-1.73
TG (54:0) / (18:0/18:0/18:0)	0.99	1.10E-06	-1.55
PI (44:4)	0.98	1.99E-06	0.97
LysoPC (16:0)	0.97	3.06E-06	-1.00
PC (38:5)	0.97	7.43E-06	-0.39
22:3 Glc-Cholesterol	0.96	1.46E-05	0.83
PC (37:2)	0.96	2.83E-05	-1.08
LysoPC (16:0e)	0.95	7.22E-05	-1.26
TG (46:0)	0.95	3.09E-04	-2.60
SM (42:2)	0.95	3.02E-05	0.84
PE (O-38:5) or PE(P-38:4)	0.95	2.09E-05	-1.42
TG (54:1)	0.95	5.63E-06	-2.03
TG (46:2)	0.94	2.93E-04	-2.69
TG (48:2)	0.94	2.16E-05	-1.62
TG (50:0)	0.94	2.57E-05	-2.22
PC (42:8) / (22:6/20:2)	0.92	2.09E-04	0.87
TG (49:5)	0.92	1.52E-04	-1.44
TG (48:1)	0.92	1.34E-04	-1.46
LysoPC (15:0)	0.91	7.65E-05	-1.04
SM (40:2)	0.90	0.0019977	0.69
PS (41:4) / (18:1/23:3)	0.90	2.90E-04	-0.28
PE (O-16:0/22:6) or PE(P-18:0/20:5)	0.90	1.01E-04	-0.62
LysoPC (18:2)	0.90	0.0013551	-0.86
PC (30:0)	0.90	0.0011812	-1.21
TG (49:3)	0.90	4.10E-04	-1.83

Serum GC-HRAM

Feature	AUC	T-tests	Log2 FC
Decanoic acid	0.99	4.33E-04	3.43
Threonic acid	0.98	1.96E-03	1.20
Proline	0.97	1.94E-06	-1.52
2,3 Biphospho-glycerate	0.97	2.82E-02	7.53
4-Hydroxyproline	0.91	1.09E-03	-1.83
Xylitol	0.85	1.30E-03	-0.98
2-Methyl Malic acid	0.85	9.26E-02	-2.62
Cysteine	0.84	2.77E-03	0.67
Indole-3-acetic acid	0.84	3.44E-02	-1.46

Table S2. Curve ROC analysis. Analysis built with significant features obtained in OD BS vs. OD comparison in serum LC-MS, serum and urine GC-HRAM-MS analyses. Features were selected with AUC values above 0.9 for LC-MS and 0.8 in GC-HRAM-MS. Abbreviations: TG, Triglycerides. DG, Diglycerides. PC, Phosphatidylcholine. LPC, Lysophosphatidylcholine. Cer, Ceramide. SM, Sphingomyelin.

Serum LC-MS

Feature	AUC	T-tests	Log2 FC
TG (50:0)	1	5.61E-06	-2.97
TG (51:1)	1	2.28E-05	-2.08
TG (52:1)	1	1.19E-07	-2.14
TG (54:1)	1	5.90E-06	-2.37
TG (54:2)	1	7.06E-06	-1.41
TG (55:1)	1	2.01E-08	-0.95
TG (50:1)	0.99	7.58E-07	-1.50
TG (56:2)	0.99	1.40E-06	-1.61
TG (52:0)	0.98	1.17E-04	-2.03
TG (55:6)	0.98	1.37E-05	-1.08
DG (32:0)	0.98	5.03E-06	-1.51
TG (50:2)	0.97	1.28E-05	-1.22
TG (54:0)	0.97	3.90E-05	-0.64
TG (36:1)	0.97	2.93E-05	-1.25
TG (46:0)	0.96	8.55E-04	-2.47
SM (42:2)	0.96	4.13E-04	0.92
PI (44:4)	0.95	1.77E-04	0.42
TG (48:1)	0.94	2.48E-04	-1.36
TG (49:5)	0.93	2.63E-04	-1.23
DG (36:3)	0.93	3.70E-04	-0.30
Cer (44:2)	0.93	3.72E-04	0.30
TG (51:2)	0.93	4.17E-04	-1.50
TG (48:2)	0.93	6.34E-04	-1.24
TG (51:7)	0.92	5.54E-04	-0.84
TG (52:2)	0.91	4.21E-04	-0.69
TG (50:3)	0.91	5.32E-04	-1.13

PC (39:0)	0.91	0.0011122	0.42
PC (36:3)	0.91	0.0019279	-0.57
TG (56:3)	0.90	3.31E-04	-1.01
TG (52:3)	0.90	8.25E-04	-0.56

Serum GC-HRAM

Feature	AUC	T-tests	Log2 FC
Glc-Cholesterol (22:3)	0.94	0.0006	0.87
Isoleucine	0.92	0.0003	-0.68
Lysine	0.91	0.0004	-0.95
Dimethylvitamin D3	0.89	0.0007	-0.75
3-Sulfogalbeta-Cer (18:1/22:0)	0.88	0.0009	-0.76
Coenzyme Q10	0.87	0.0053	0.63
N-Carboxyglycine	0.83	0.0072	-1.01
Galactose	0.81	0.0146	-0.71
Serine	0.80	0.0309	-1.10
Valine	0.80	0.0197	-0.54
Isocaproic acid	0.80	0.0081	-0.47

Urine GC-HRAM

Feature	AUC	T-tests	Log2 FC
Decanoic acid	0.91	0.0021	0.94
Tridecanoic acid	0.91	0.0167	-0.97
Caffeine	0.86	0.0296	-1.44
Ureidopropionic acid	0.86	0.0078	-1.07
Hippuric acid	0.83	0.0193	0.85
2-hydroxyphenyl-acetic acid	0.81	0.0867	-0.25

Figure S1. Lipidomic pathway for discriminating features found in serum between OD and O patients. Font colors (yellow and green) represent up- or down- regulated lipids with significant changes in OD patients, in comparison with O group. Criteria for the selection of the most rele-vant compounds: features were considered statistically significant when reached a p-value of ≤ 0.05 or a VIP value of > 1. Therefore, UVA and MVA statistical results were used to select the compounds with the highest biological relevance. P-values and VIP values were included for each significant feature in Table S4. Abbreviations: TG, Triglycerides. DG, Diglycerides. PC, Phospha-tidylcholine. LPC, Lysophosphatidylcholine. Cer, Ceramide. SM, Sphingomyelin. PA, Phospha-tidic acid. PI, Phosphatidylinositol. LPI, Lysophosphatidylinositol. PE, Phosphatidylethanolamine. LPE, Lyso phosphatidylethanolamine. PS, Phosphatidylserine. LPS, Lysophosphatidylserine. PE-Me, Phosphatidylethanolamine N-methyl.

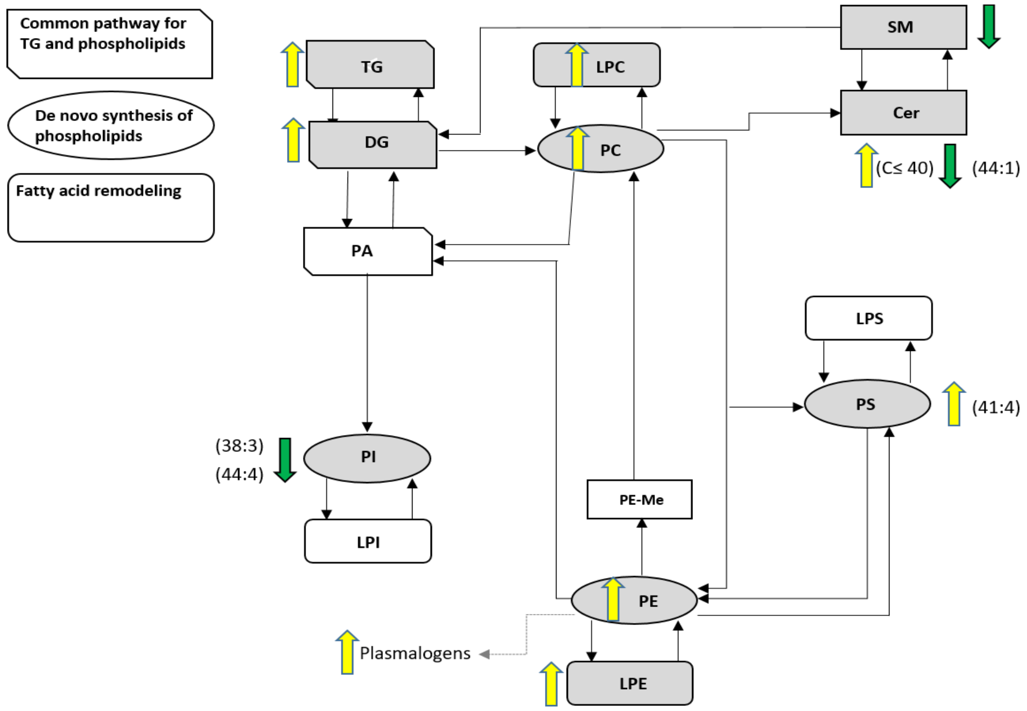

Figure S2. Amino acid pathway for discriminating features found in serum between OD and O patients. Font colors (yellow and green) represent up- or down- regulated lipids with remarka-ble changes in OD patients, in comparison with O group. Significant features were illustrated in full boxes, while non-significant features were in dash boxes. Black arrows represent anabolic routes and red arrows, catabolic routes. Criteria for the selection of the most relevant compounds: features were considered statistically significant when reached a p-value of ≤ 0.05 or a VIP value of > 1. Therefore, UVA and MVA statistical results were used to select the compounds with the highest biological relevance. P-values and VIP values were included for each significant feature in Table S4. Abbreviations: Per, Peroxisome. Mit, Mitochondrial. Ile, isoleucine. Cys, Cysteine. Tyr, Tyrosine. Arg, Arginine. Pro, Proline.

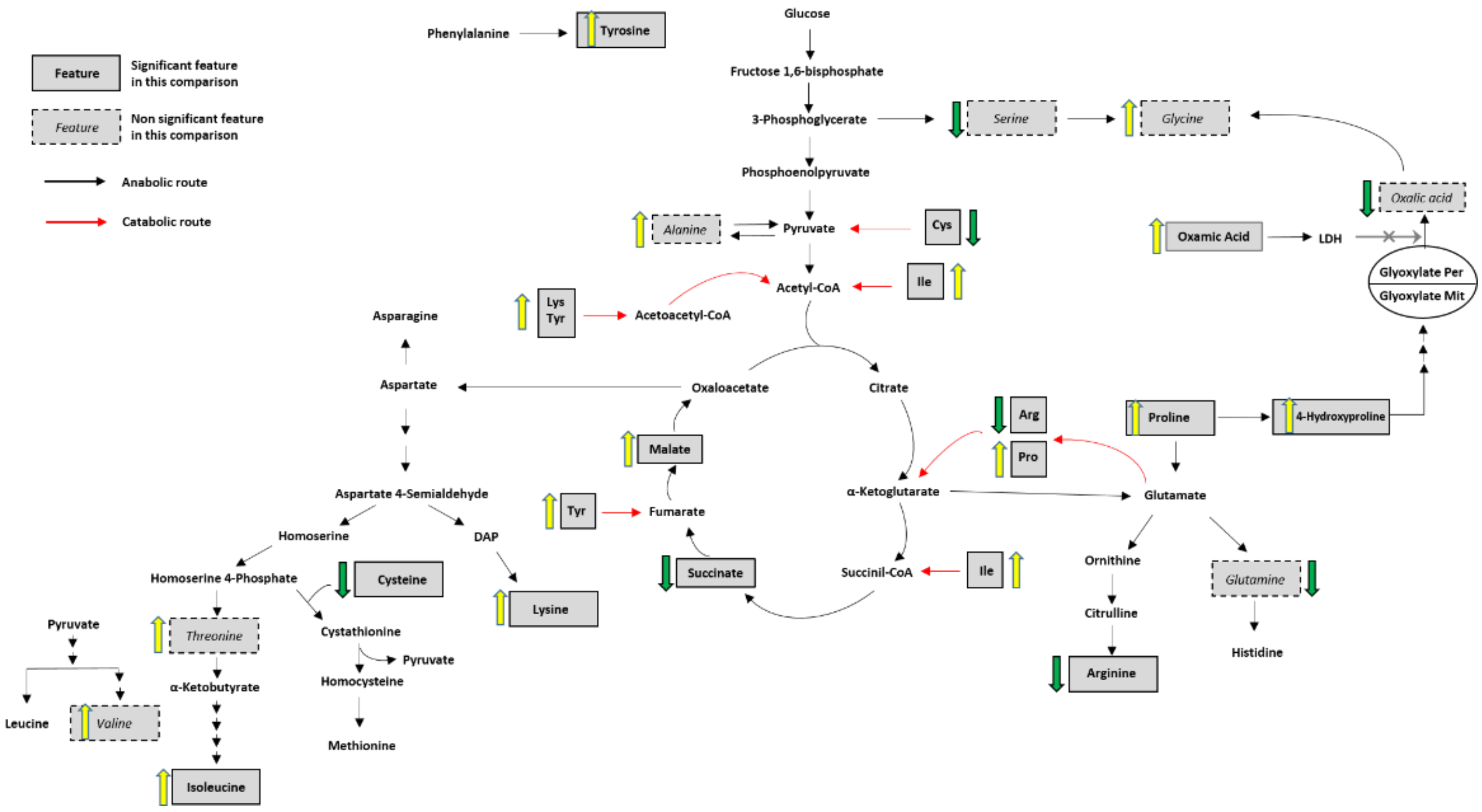

Figure S3. Lipidomic pathway for discriminating features found in serum between OD BS and OD patients. Font colors (yellow and green) represent up- or down- regulated lipids with signifi-cant changes in OD BS patients, in comparison with OD group. Criteria for the selection of the most relevant compounds: features were considered statistically significant when reached a p-value of ≤ 0.05 or a VIP value of > 1. Therefore, UVA and MVA statistical results were used to select the compounds with the highest biological relevance. P-values and VIP values were in-cluded for each significant feature in Table S4. Abbreviations: TG, Triglycerides. DG, Diglycerides. PC, Phosphatidylcholine. LPC, Lysophosphatidylcholine. Cer, Ceramide. SM, Sphingomyelin. PA, Phosphatidic acid. PI, Phosphatidylinositol. LPI, Lysophosphatidylinositol. PE, Phosphatidyleth-anolamine. LPE, Lyso phosphatidylethanolamine. PS, Phosphatidylserine. LPS, Lysophosphati-dylserine. PE-Me, Phosphatidylethanolamine N-methyl.

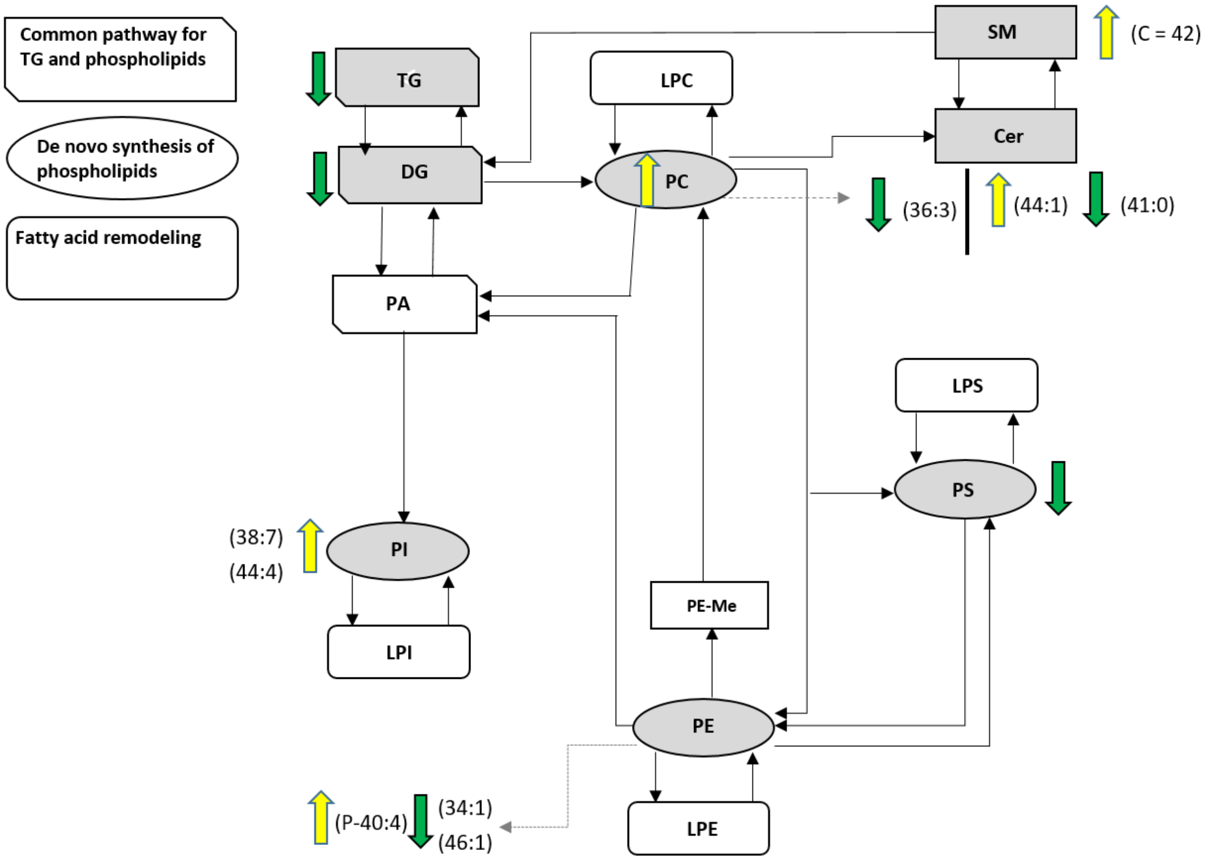

Figure S4. Amino acid pathway for discriminating features found in serum between OD BS and OD patients. Font colors (yellow and green) represent up- or down- regulated lipids with re-markable changes in OD BS patients, in comparison with OD group. Significant features were il-lustrated in full boxes, while non-significant features were in dash boxes. Black arrows represent anabolic routes and red arrows, catabolic routes. Criteria for the selection of the most relevant compounds: features were considered statistically significant when reached a p-value of ≤ 0.05 or a VIP value of > 1. Therefore, UVA and MVA statistical results were used to select the compounds with the highest biological relevance. P-values and VIP values were included for each significant feature in Table S4. Abbreviations: Per, Peroxisome. Mit, Mitochondrial. Ala, Alanine. Gly, Gly-cine. Ser, Serine. Thre, Threonine. Ile, Isoleucine. Lys, Lysine. Gln, Glutamine. Pro, Proline.

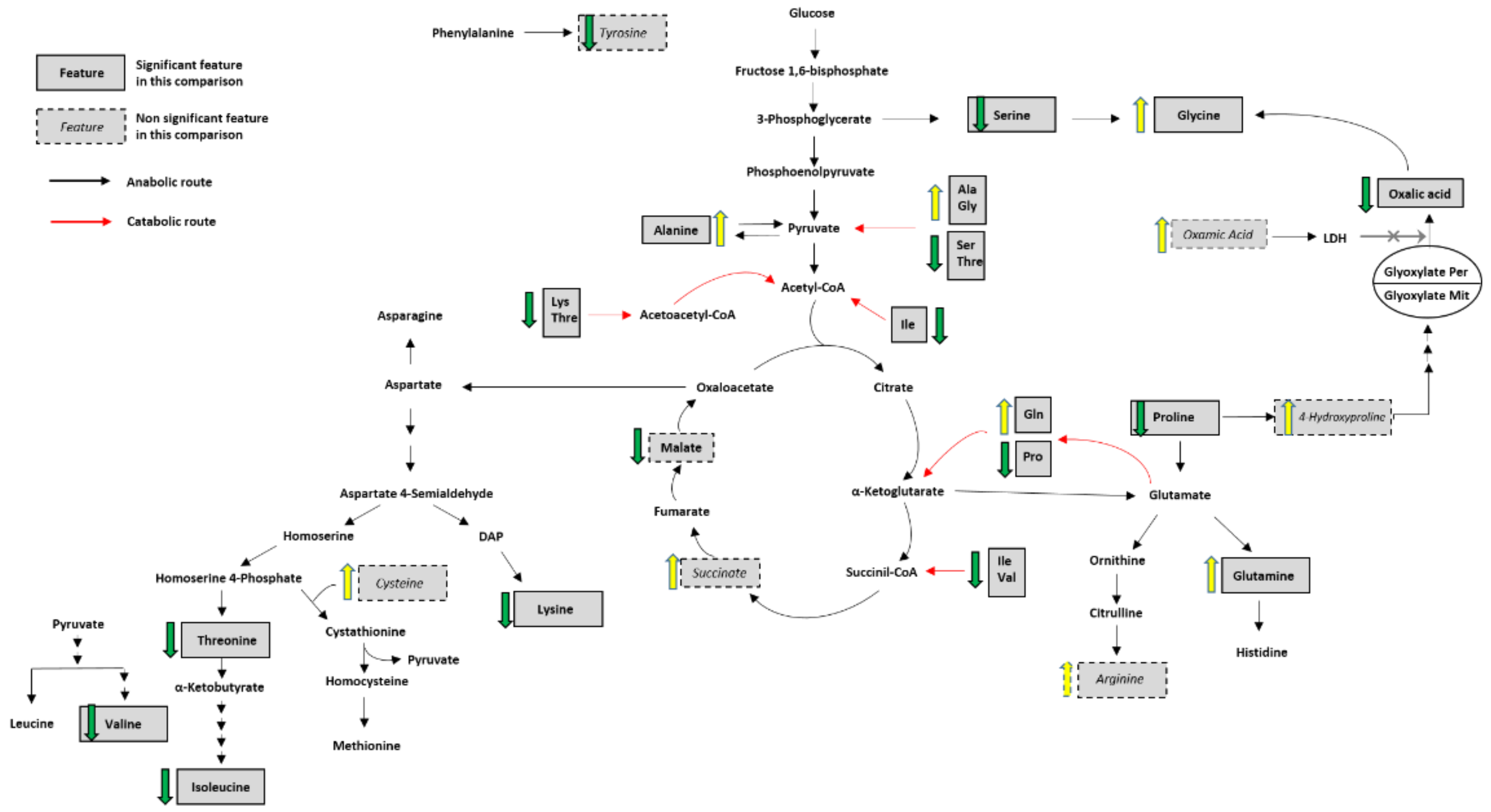

Table S3. Detailed significant correlations obtained for OD, OD BS and O patients compared with clinical parameters highly related to kidney dysfunction in significant features found in serum LC-MS for OD vs. O and OD BS vs. OD comparisons. Abbreviations: TG, Triglycerides. DG, Diglycerides. PC, Phosphatidylcholine. LPC, Lysophosphatidylcholine. Cer, Ceramide. PE, Phosphatidylethanolamine. PS, Phosphatidylserine. PI, Phosphatidylinositol. SM, Sphingomyelin.

Glucose

	O		OD		OD BS	
**Feature**	**p-value**	**Correlation**	**p-value**	**Correlation**	**p-value**	**Correlation**
Cer (d36:1)	0.00008	0.86				
Cer (d40:1) / (18:1/22:0)	0.00030	0.82				
Cer (41:0)	0.00193	0.75				
Cer (44:1)	0.00016	-0.84				
LysoPE(18:1)	0.00111	0.78				
PE (O-38:5) or PE(P-38:4)	0.03506	0.57				
PE (34:1) / (16:0/18:1)	0.00001	0.90				
PE (46:1)	0.00003	0.88				
LysoPC (16:0)	0.03432	0.57				
LysoPC (18:1)	0.02109	0.61				
LysoPC (20:4)	0.04491	0.54				
PC (O-42:5)			0.042	-0.619		
DG (32:0)	0.00034	0.82				
DG (32:1) / (14:0/18:1)	0.00085	0.79				
DG (34:1)	0.00028	0.83				
DG (36:1)	0.00004	0.88				
DG (36:3)	0.00000	0.95				
DG (36:3) / (18:1/18:2)	0.00045	0.81				
DG (36:4) /(16:1/20:3)	0.00138	0.77				
TG (46:0)	0.00006	0.87				
TG (46:2)	0.00004	0.88				
TG (48:1)	0.00038	0.81				
TG (48:2)	0.00013	0.85				
TG (48:3)	0.02188	0.61				
TG (49:3)	0.00002	0.89				
TG (49:5)	0.00037	0.82				
TG (50:0)	0.00000	0.92				
TG (50:1)	0.00003	0.88				
TG (50:2) //(14:0/18:1/18:1)	0.00001	0.90				
TG (50:3)	0.00001	0.91				
TG (51:1)	0.00005	0.87				
TG (51:2)	0.00002	0.89				
TG (51:7)	0.00022	0.83				
TG (52:0)	0.00001	0.90				
TG (52:1) / (16:0/18:0/18:1)	0.00001	0.90				
TG (52:2) / (18:1/18:1/16:0)	0.00018	0.84				
TG (52:3) / (18:2/18:1/16:0)	0.00012	0.85				
TG (52:4) / (16:0/18:2/18:2)	0.00004	0.88				
TG (52:5) / (16:0/18:2/18:3)	0.00002	0.89				
TG (53:3)	0.00055	0.80				
TG (54:0) / (18:0/18:0/18:0)	0.00000	0.93				
TG (54:1)	0.00001	0.90				
TG (54:2)	0.00001	0.90				
TG (54:3) //(18:1/18:1/18:1)	0.00008	0.86				
TG (54:4) //(18:2/18:1/18:1)	0.00004	0.87				
TG (54:5) //(18:1/18:2/18:2)	0.00013	0.85				
TG (54:6)	0.00014	0.85			0.01381	0.71
TG (55:1)	0.00002	0.89				
TG (55:6)	0.00010	0.85				
TG (56:2)	0.00000	0.92				
TG (56:3)	0.00002	0.89				
TG (56:4)	0.00004	0.88				

Cholesterol

	O		OD		OD BS	
Feature	p-value	Correlation	p-value	Correlation	p-value	Correlation
3-demethylubiquinone-9					0.015	0.71
22:3 Glc-Cholesterol					0.023	0.67
3"-Sulfogalbeta-Cer (d18:1/22:0)	0.011	0.68				
Cer (d40:1) / (18:1/22:0)	0.053	0.55			0.042	0.62
Cer (41:0)	0.030	0.60	0.041	0.62	0.010	0.73
Cer (44:1)	0.035	-0.59				
SM (37:1) / (18:1/19:0)					0.044	0.61
SM (d39:1) / (18:1/21:0)	0.037	0.58				
SM (40:2)					0.010	0.74
SM (d41:1)	0.016	0.65			0.002	0.82
SM (42:2)					0.011	0.73
SM (d42:3) /(d18:2/24:1)			0.022	0.68		
beta-hydroarchaetidylglycerol	0.006	0.72			0.009	0.74
PI (38:3) / (18:0/20:3)					0.023	0.67
PI (44:4)					0.044	0.62
PS (39:6) / (19:0/20:6)	0.020	0.64				
PS (41:5) / (18:0/23:5)	0.006	0.71				
PS (41:6) / (18:1/23:5)	0.038	0.58				
PE (O-16:0/22:6) or PE(P-18:0/20:5)	0.012	0.67				
LysoPC (15:0)			0.029	0.65		
LysoPC (18:0)					0.033	0.64
LysoPC (20:3)					0.026	0.66
LysoPC (20:4)						
PC (30:0)			0.013	0.71		
PC (35:2)	0.015	0.66				
PC (35:3) / (18:2/17:1)	0.010	0.69				
PC (36:2)	0.003	0.76				
PC (36:3)	0.003	0.75				
PC (38:5)	0.034	0.59				
PC (42:8) / (22:6/20:2)					0.003	0.81
DG (32:1) / (14:0/18:1)			0.019	0.69		
DG (36:3) / (18:1/18:2)	0.040	0.57				
DG (36:4) /(16:1/20:3)	0.047	0.56			0.035	0.64
TG (48:1)			0.041	0.62		
TG (48:3)			0.037	0.63		
TG (48:4)					0.033	0.64
TG (49:3)			0.003	0.80		
TG (49:5)			0.039	0.63		
TG (50:3)			0.024	0.67		
TG (51:1)			0.048	0.61		
TG (51:2)			0.025	0.67		
TG (51:7)			0.028	0.66		
TG (54:5) //(18:1/18:2/18:2)	0.049	0.55				
TG (54:6)	0.038	0.58				

LDL

	O		OD		OD BS	
**Feature**	**p-value**	**Correlation**	**p-value**	**Correlation**	**p-value**	**Correlation**
3-demethylubiquinone-9					0.045	0.61
22:3 Glc-Cholesterol					0.048	0.61
Dimethylvitamin D3 or Cholesteryl acetate	0.034	-0.64				
3"-Sulfogalbeta-Cer (d18:1/22:0)	0.040	0.63				
Cer (41:0)					0.017	0.70
SM (40:2)					0.038	0.63
SM (d41:1)			0.048	0.71	0.009	0.74
SM (42:2)					0.028	0.66
SM (d42:3) /(d18:2/24:1)			0.015	0.81		
beta-hydroarchaetidylglycerol					0.021	0.68
PS (41:4) / (18:1/23:3)			0.010	-0.84		
PS (41:5) / (18:0/23:5)	0.040	0.62				
LysoPC (14:0)			0.010	0.84		
LysoPC (15:0)			0.000	0.98		
LysoPC (16:0)			0.003	0.89		
LysoPC (18:0)			0.016	0.80		
LysoPC (18:1)			0.010	0.83		
LysoPC (20:3)			0.048	0.71		
LysoPC (20:4)			0.033	0.75		
PC (36:3)	0.021	0.68				
PC (38:5)			0.008	-0.85		
PC (42:8) / (22:6/20:2)					0.019	0.69
DG (36:4) /(16:1/20:3)					0.031	0.65
TG (48:3)	0.024	0.67				
TG (56:4)			0.026	-0.77		

Uric acid

	O		OD		OD BS	
**Feature**	**p-value**	**Correlation**	**p-value**	**Correlation**	**p-value**	**Correlation**
Coenzyme Q9	0.014	0.68				
SM (d39:1) / (18:1/21:0)	0.031	0.62				
beta-hydroarchaetidylglycerol	0.041	0.60				
LysoPC (14:0)					0.023	-0.67
LysoPC (15:0)	0.015	0.68				
LysoPC (18:0)	0.012	0.69				
LysoPC (18:2)	0.032	0.62				
LysoPC (20:3)	0.010	0.71				
PC (35:2)	0.011	0.70				
PC (35:3) / (18:2/17:1)	0.014	0.69				
PC (36:2)	0.029	0.63				
PC (37:2)	0.030	0.62				
PC (38:2) / (20:1/18:1)	0.029	0.63				
DG (32:0)					0.039	0.63
DG (36:1)					0.024	0.67
DG (36:3) / (18:1/18:2)					0.002	0.81
DG (36:4) /(16:1/20:3)					0.006	0.76
TG (46:0)			0.031	0.649		
TG (46:2)			0.022	0.676		
TG (48:1)			0.041	0.621		
TG (49:5)			0.043	0.617		
TG (50:0)					0.010	0.74
TG (52:0)					0.030	0.65
TG (52:1) / (16:0/18:0/18:1)					0.030	0.65
TG (52:3) / (18:2/18:1/16:0)					0.035	0.64
TG (52:4) / (16:0/18:2/18:2)					0.049	0.60
TG (55:6)					0.046	0.61

Creatinine

	O		OD		OD BS	
**Feature**	**p-value**	**Correlation**	**p-value**	**Correlation**	**p-value**	**Correlation**
22:3 Glc-Cholesterol	0.007	-0.68				
Cer (44:1)	0.025	-0.59				
SM (40:2)	0.038	-0.56				
SM (42:2)	0.011	-0.66				
PI (44:4)	0.007	-0.68				
PE (34:1) / (16:0/18:1)			0.035	0.637		
LysoPC (14:0)	0.039	0.56			0.039	-0.627
LysoPC (18:1)	0.045	0.54				
PC (36:0) / (18:0/18:0)	0.002	-0.76				
PC (36:2)			0.019	0.690		
PC (36:4) /(18:2/18:2)					0.029	0.654
PC (39:0)	0.010	-0.66				
DG (36:3)	0.039	0.56				
TG (46:0)			0.006	0.762		
TG (46:2)			0.011	0.730		
TG (48:1)			0.025	0.665		
TG (48:2)	0.046	0.54	0.025	0.667		
TG (48:4)	0.023	-0.60				
TG (49:3)	0.020	0.61				
TG (49:5)			0.027	0.660		
TG (50:0)			0.006	0.768		
TG (50:3)	0.023	0.60				
TG (51:7)	0.019	0.62				
TG (52:0)			0.045	0.614		
TG (52:1) / (16:0/18:0/18:1)			0.043	0.617		
TG (52:4) / (16:0/18:2/18:2)	0.027	0.59				
TG (52:5) / (16:0/18:2/18:3)	0.017	0.62				
TG (54:5) //(18:1/18:2/18:2)	0.050	0.53				
TG (55:1)			0.039	0.626		

eGFR

	O		OD		OD BS	
**Feature**	**p-value**	**Correlation**	**p-value**	**Correlation**	**p-value**	**Correlation**
3-demethylubiquinone-9	0.001	0.78				
Coenzyme Q9	0.033	0.57				
SM (d39:1) / (18:1/21:0)	0.020	0.61				
PE (O-38:5) or PE(P-38:4)					0.046	-0.61
PE (34:1) / (16:0/18:1)			0.016	-0.70		
PC (36:0) / (18:0/18:0)	0.016	0.63				
PC (36:2)			0.006	-0.77		
PC (36:3)						
PC (36:4) /(18:2/18:2)					0.010	-0.74
PC (39:0)	0.009	0.67				
TG (46:0)			0.020	-0.69		
TG (46:2)			0.037	-0.63		
TG (48:1)			0.045	-0.61		
TG (49:5)			0.048	-0.61		
TG (50:0)			0.049	-0.60		

Proteinuria

	O		OD		OD BS	
**Feature**	**p-value**	**Correlation**	**p-value**	**Correlation**	**p-value**	**Correlation**
Dimethylvitamin D3 or Cholesteryl acetate	0.008	0.68				
SM (40:2)	0.008	0.68				
PC (35:2)			0.007	0.75		
PC (35:3) / (18:2/17:1)			0.032	0.65		
PC (38:1) / (20:1 / 18:0)	0.004	0.71				
PC (38:2) / (20:1/18:1)			0.030	0.65		
PC (O-42:5)	0.038	0.56				
DG (36:3) / (18:1/18:2)			0.044	0.61		
DG (36:4) /(16:1/20:3)			0.046	0.61		
TG (52:4) / (16:0/18:2/18:2)			0.012	0.72		
TG (52:5) / (16:0/18:2/18:3)			0.001	0.84		
TG (53:3)			0.003	0.81		
TG (54:0) / (18:0/18:0/18:0)			0.044	0.61		

UACR

	O		OD		OD BS	
**Feature**	**p-value**	**Correlation**	**p-value**	**Correlation**	**p-value**	**Correlation**
Coenzyme Q10	0.012	0.75				
22:3 Glc-Cholesterol	0.032	0.68				
Dimethylvitamin D3 or Cholesteryl acetate	0.002	0.84				
SM (d36:2)	0.046	0.64				
SM (37:1) / (18:1/19:0)			0.027	0.69		
SM (d38:2)	0.009	0.77				
SM (40:2)	0.004	0.81				
SM (42:2)	0.038	0.66				
SM (d42:3) /(d18:2/24:1)	0.004	0.82				
PI (38:3) / (18:0/20:3)			0.044	0.65		
PE (P-40:4) / (O-40:5)			0.018	0.72		
PC (38:1) / (20:1 / 18:0)	0.001	0.89				
PC (O-42:5)	0.016	0.73				
PC (42:8) / (22:6/20:2)	0.044	0.64				
PC (44:5) / (20:4/24:1)	0.019	0.72				
TG (48:4)	0.008	0.78				

Figure S5. Unsupervised PCA model: features of OD, OD BS and O patients obtained in serum for LC-MS analysis were analyzed in a multivariate statistical analysis (MVA) performed in SIMCA-P 16.0. White triangles, O patients; black circles, OD patients; soft grey squares, OD BS patients. Analyzing all the lipids simultaneously obtained after RSD filtration through a PCA model, the lipidomic profiles between obese patients with or without CKD were different. The samples corresponding to OD and OD BS patients were perfectly separated in the ellipse by the primary variable (vertical) and the second variable (horizontal). Bariatric surgery in patients with CKD induced changes in their lipidome that allowed these patients to approach the position of obese patients without kidney disease (O) in the ellipse. These changes in the individuals after bariatric surgery practically eliminated the variable that allowed them to separate vertically from obese patients without kidney disease before the intervention.

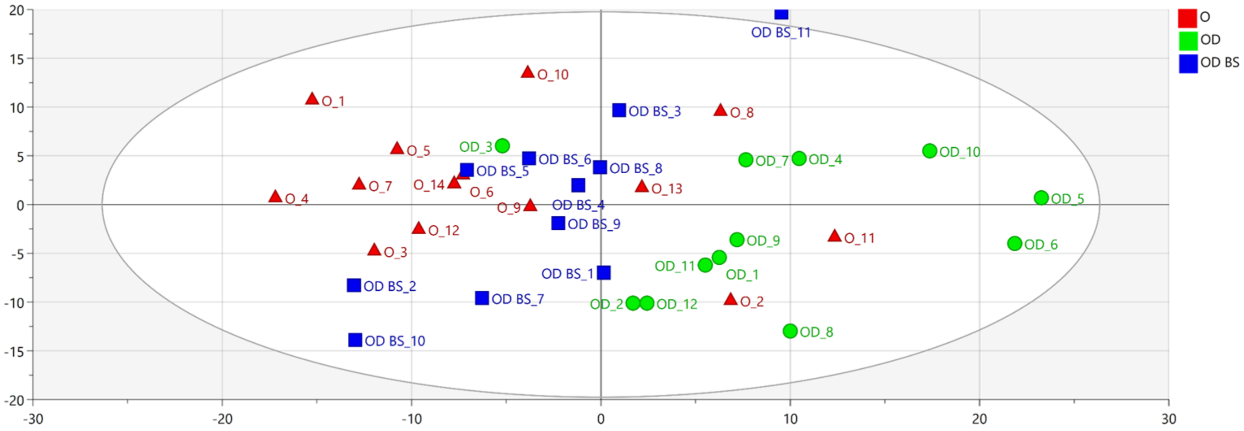

Figure S6. OPLS-DA model yield between OD and O patients with serum analyzed by LC-MS. Variables were scaled with UV (unit variance). R2Y, fit, and Q2, predictive ability, were included for model diagnosis. Validation was performed with CV ANOVA tool and the resulting P value was showed. O patients red-triangles; OD patients, green-circles.

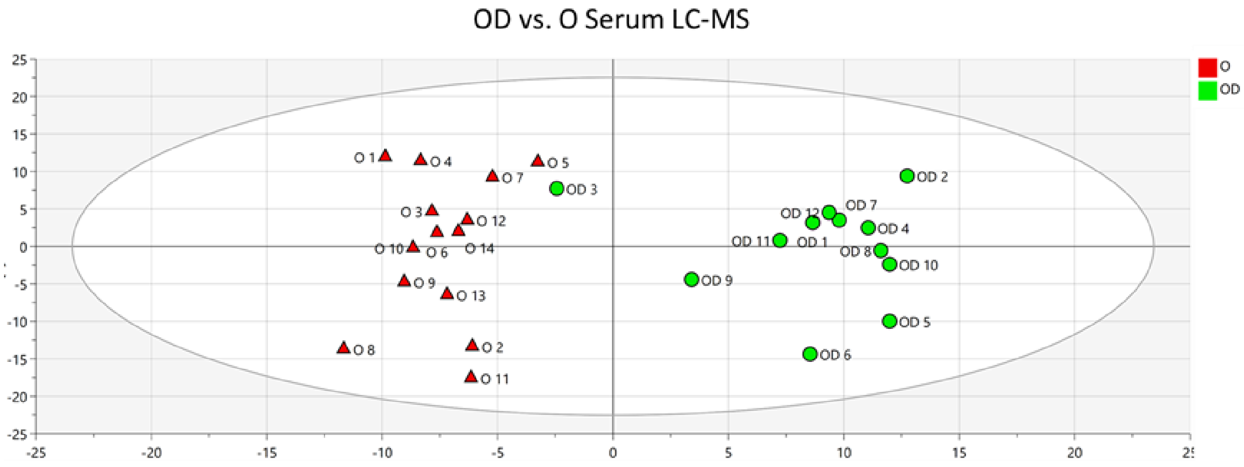

R^2Y	Q^2	CV ANOVA
0.863	0.523	0.0027

Figure S7. OPLS-DA model yield between OD and O patients with serum analyzed by GC-HRAM-MS. Variables were scaled with pareto (unit variance) and presented a logarithmic transformation. R2Y, fit, and Q2, predictive ability, were included for model diagnosis. Validation was performed with CV ANOVA tool and the resulting P value was showed. O patients red-triangles; OD patients, green-circles.

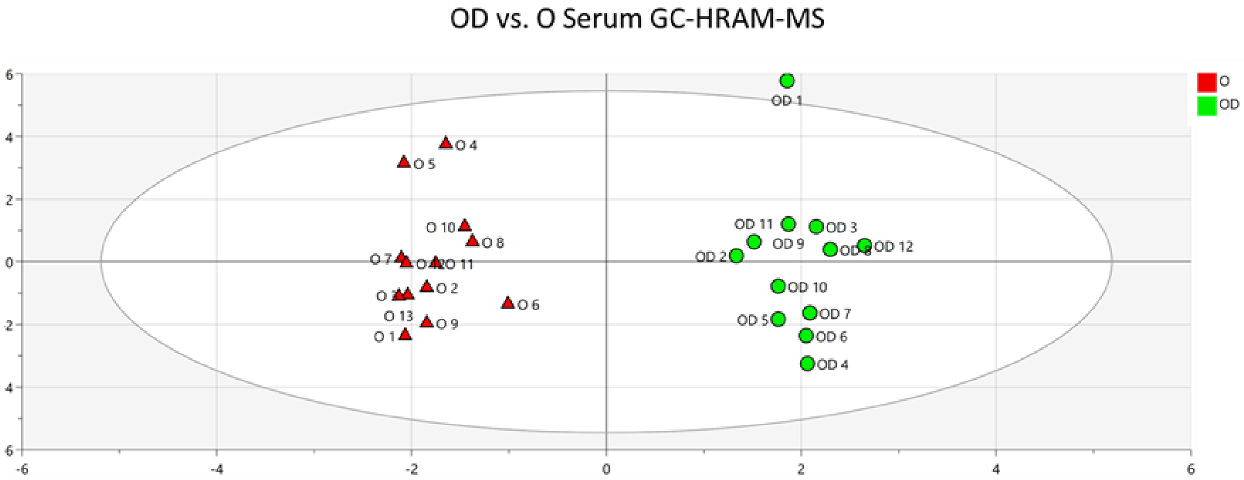

R^2Y	Q^2	CV ANOVA
0.969	0.778	4.64E-05

Figure S8. OPLS-DA model yield between OD BS and OD patients with serum analyzed by LC-MS. Variables were scaled with UV (unit variance). R2Y, fit, and Q2, predictive ability, were included for model diagnosis. Validation was performed with CV ANOVA tool and the resulting P value was showed. OD patients, green-circles; OD BS patients, blue-boxes.

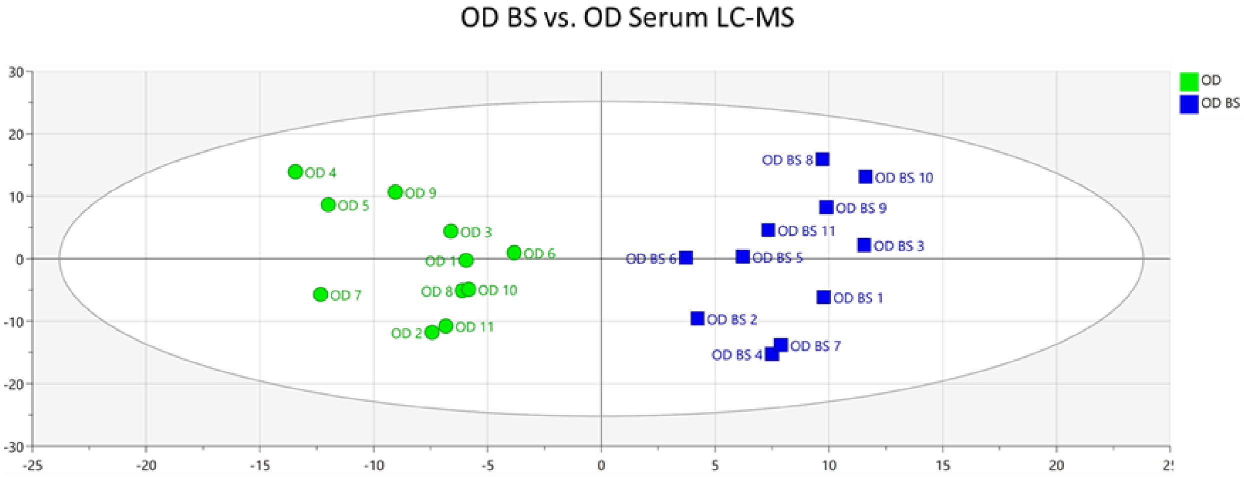

R²Y	Q²	CV ANOVA
0.895	0.633	0.0012

Figure S9. OPLS-DA model yield between OD BS and OD patients with serum analyzed by GC-HRAM-MS. Variables were scaled with UV (unit variance). R2Y, fit, and Q2, predictive ability, were included for model diagnosis. Validation was performed with CV ANOVA tool and the resulting P value was showed. OD patients, green-circles; OD BS patients, blue-boxes.

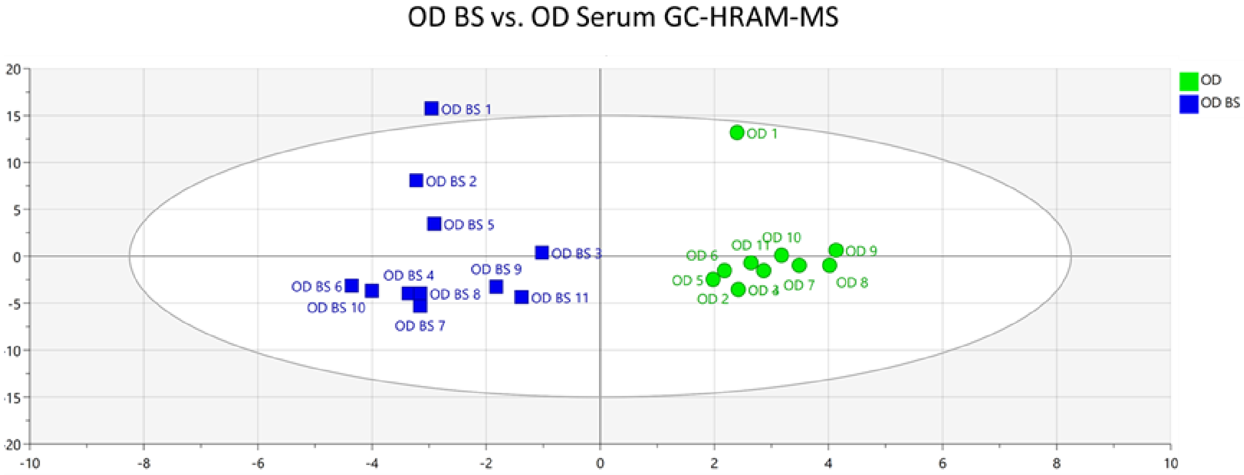

R²Y	Q²	CV ANOVA
0.914	0.36	0.2752

Figure S10. OPLS-DA model yield between OD BS and OD patients with urine analyzed by GC-HRAM-MS. Variables were scaled with centroid and presented a logarithmic transformation. R2Y, fit, and Q2, predictive ability, were included for model diagnosis. Validation was performed with CV ANOVA tool and the resulting P value was showed. OD patients, green-circles; OD BS patients, blue-boxes.

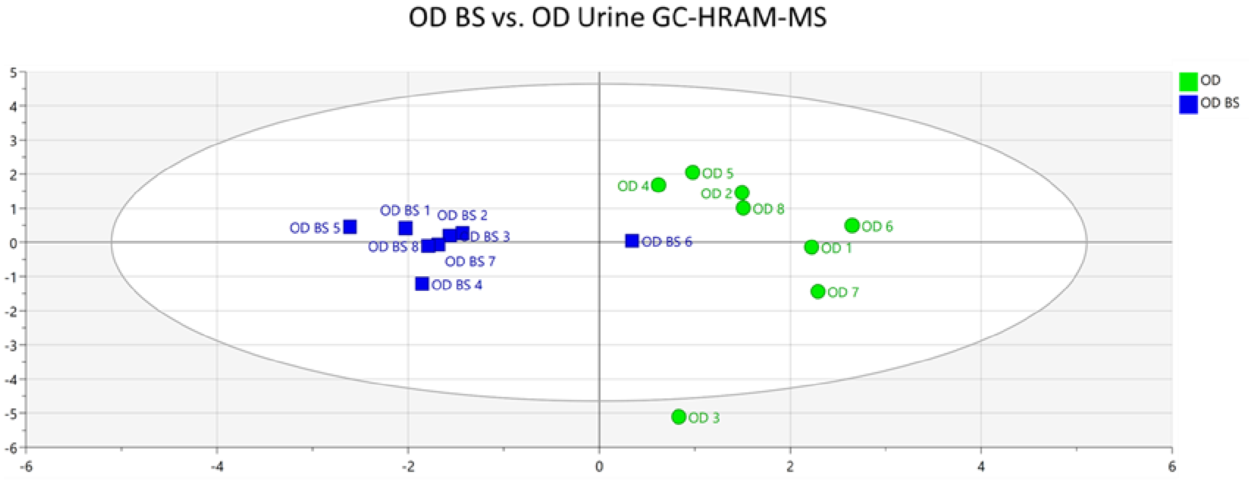

R^2Y	Q^2	CV ANOVA
0.815	0.448	0.1322

Table S4. Statistically significant metabolites found in UVA and MVA statistical analysis performed in OD vs. O and OD BS vs. OD comparisons.

Serum significant features annotated in LC-MS and GC-HRAM-MS analyses.

Compound	Mass	RT (min)	RI Exp	RI DB	CV (% in QC)	Adduct	ID source	Analytical platform	OD vs. O			OD BS vs. OD		
									FC	p-value	OPLS-DA VIP	FC	p-value	OPLS-DA VIP
**Carboxylic acids and derivatives**														
**Amino acids, peptides, and analogues**														
2-amino Butanoic acid	103.0633	9.55	1181.34	1179.47	8.3		GMD	GC-HRAM	0.71	0.03201	0.88	0.68	0.05863	1.20
3-amino Isobutanoic acid	103.0633	13.74	1453.76	1455.34	22.2		GMD	GC-HRAM				1.47	0.04936	1.26
4-Hydroxyproline	131.0582	14.65	1522.71	1524.11	33.3		GMD	GC-HRAM	3.33	0.00680	1.97			
Alanine	89.0476	12.50	1363.30		25.2		In-house library	GC-HRAM				1.27	0.17273	0.91
Aminomalonic acid	119.0218	13.93	1467.81	1479	32.3		NIST	GC-HRAM	1.17	0.02399	0.97			
Arginine	174.1116	18.01	1812.73	1813.78	24.0		GMD	GC-HRAM	0.67	0.00810	1.17			
Cysteine	121.0197	15.01	1552.55		39.9		In-house library	GC-HRAM	0.62	0.00140	1.21			
Glutamine	146.0691	13.99	1471.77	1468.99	35.6		GMD	GC-HRAM				1.46	0.02079	1.35
Glycine	75.032	11.68	1302.41	1302.68	4.5		GMD	GC-HRAM				1.20	0.04385	1.19
Isoleucine	131.0946	11.19	1275.22	1280.12	3.6		GMD	GC-HRAM	1.28	0.02388	0.82	0.63	0.00315	2.05
Lysine	146.1055	18.37	1846.54	1849.23	36.8		GMD	GC-HRAM	1.33	0.12459	1.15	0.57	0.00061	1.65
N-Carboxyglycine	119.08	13.87	1463.62	1462.28	39.3		GMD	GC-HRAM				0.54	0.02907	1.46
Norvaline	117.0789	10.26	1221.81	1229.91	38.8		GMD	GC-HRAM	1.49	0.02414	1.05	0.78	0.09080	1.05
Proline	115.0633	11.55	1294.72		24.5		In-house library	GC-HRAM	2.72	0.00007	2.03	0.59	0.03450	0.93
Serine	105.0425	12.32	1350.16		35.1		In-house library	GC-HRAM				0.51	0.02058	1.24
Threonine	119.1192	12.84	1387.45		7.4		In-house library	GC-HRAM				0.80	0.09568	1.02
Tyrosine	181.0738	19.27	1933.19		38.5		In-house library	GC-HRAM	1.43	0.07742	0.90			
Valine	117.0789	10.28	1223.19		2.2		In-house library	GC-HRAM				0.66	0.04105	1.53
**Dicarboxylic acids and derivatives**														
Oxalic acid	89.9953	8.41	1115.70	1118.32	24.2		GMD	GC-HRAM				0.69	0.10076	1.20
Oxamic acid	89.05	10.85	1256.39	1255.25	22.2		GMD	GC-HRAM	1.40	0.01798	1.06			
Succinic acid	118.0266	11.80	1311.61	1310.65	19.7		GMD	GC-HRAM	0.87	0.02079	0.60			
**Fatty Acyls**														
**Fatty acids and conjugates**														
9-Octadecenoic acid	282.2558	21.92	2230.30	2225.44	30.7		GMD	GC-HRAM	0.72	0.05349	1.08			
Decanoic acid	172.268	13.82	1459.55		32.9		In-house library	GC-HRAM	0.09	0.00000	2.94			
Ethyl Malonic acid	132.0422	11.08	1269.36	1273.31	23.6		GMD	GC-HRAM	1.49	0.03599	1.13			
Isocaproic acid	116.0837	10.01	1206.85	1206.44	4.9		GMD	GC-HRAM				0.73	0.00903	1.64
Stearic Acid	284.2715	22.16	2261.15	2236	33.9		Fiehn	GC-HRAM				0.72	0.04858	1.40
**Homogeneous other non-metal compounds**														
Hydroxylamine	33.0214	8.34	1111.37	1105	9.3		Fiehn	GC-HRAM				1.45	0.03053	1.33
**Hydroxy acids and derivatives**														
**Beta hydroxy acids and derivatives**														
2-Methyl Malic acid	148.11	13.87	1463.35	1464.37	37.9		GMD	GC-HRAM	5.65	0.00408	2.10	2.30	0.19426	0.95
Malic acid	134.0215	14.05	1475.92	1477.88	20.3		GMD	GC-HRAM	1.40	0.09876	1.08			

**Indoles and derivatives**														
**Indolyl carboxylic acids and derivatives**														
Indole-3-acetic acid	175.0633	19.51	1957.41		39.0		In-house library	GC-HRAM	2.72	0.00361	1.54	1.82	0.02196	0.85
**Non-metal oxoanionic compounds**														
**Non-metal sulfites**														
Sulfonic acid	81.9724	9.07	1155.25	1153.97	7.7		GMD	GC-HRAM				1.96	0.11928	1.22
**Organic phosphoric acids and derivatives**														
**Phosphate esters**														
Methanolphosphate	111.9925	9.65	1186.53	1167	39.8		Fiehn	GC-HRAM				0.71	0.03774	1.21
**Organonitrogen compounds**														
**Amines**														
Ethanolamine	61.0527	13.48	1434.51	1433.85	3.1		GMD	GC-HRAM	1.38	0.02816	1.01			
**Organooxygen compounds**														
**Carbohydrates and carbohydrate conjugates**														
2,3 Biphospho-glycerate	265.9592	13.16	1411.07	1396	21.7		Fiehn	GC-HRAM	0.01	0.00008	4.70			
2-amino,2-deoxy Gluconic acid	699.3509	20.15	2023.51	2025.73	17.8		GMD	GC-HRAM				0.74	0.06156	1.28
Arabitol	152.0684	16.93	1712.20		18.2		In-house library	GC-HRAM				0.85	0.05971	0.61
Galactose	180.0633	19.02	1906.44	1902.42	32.0		GMD	GC-HRAM				0.67	0.03632	1.49
Glycerol	92.0473	11.05	1267.66	1264.6	30.5		GMD	GC-HRAM	0.74	0.06058	1.00			
Mannitol	182.079	19.07	1911.81	1913.17	21.6		GMD	GC-HRAM				1.48	0.05259	1.24
Threonic acid	136.0371	15.08	1557.98	1545	11.7		Fiehn	GC-HRAM	0.49	0.00023	1.48			
Xylitol	152.0684	16.73	1694.16	1694.5	26.5		GMD	GC-HRAM	1.88	0.00617	1.29	0.81	0.04168	0.60
**Prenol Lipids**														
**Quinones and hydroquinones**														
3-demethylubiquinone-9	780.6106	7.01			1.7	M+H	MS-MS	LC-MS (+)	0.79	0.01080	1.20			
Coenzyme Q10	862.6839	6.92			2.8	M+K	MS-MS	LC-MS (+)	0.64	0.00262	1.44	1.39	0.00256	1.44
**Saturated hydrocarbons**														
**Alkanes**														
Nonadecane	268.313	18.95	1898.98	1900	6.8		GMD	GC-HRAM				1.20	0.04404	1.06
**Sterol Lipids**														
**Sterols**														
22:3 Glc-Cholesterol	902.646422	7.31			0.6	M+H	MS-MS	LC-MS (+)	0.63	0.00109	1.56	1.69	0.00274	1.63
Dimethylvitamin D3 or 1-Hydroxyvitamin D5 or Cholesteryl acetate	428.364821	5.43			12.4	M+H	MS-MS	LC-MS (+)				0.61	0.00455	1.51
**Sphingolipids**														
**Acidic glycosphingolipids**														
3"-Sulfogalbeta-Cer (d18:1/22:0)	879.6105	6.57			18.0	M+Na	MS-MS	LC-MS (+)				0.58	0.00190	1.39
**Ceramides**														
Cer (d36:1)	581.5383	8.55			6.1	M+Na	MS-MS	LC-MS (+)	1.64	0.00280	1.48			

Cer (d40:1) / (18:1/22:0)	621.6059	7.65			14.4	M-H2O	MS-MS	LC-MS (+)	1.52	0.00713	1.39			
Cer (41:0)	635.621266	7.81			5.1	-	In-house library	LC-MS (+)				0.60	0.00619	1.31
Cer (44:1)	661.6737	8.31			23.3	M+K	MS-MS	LC-MS (+)	0.56	0.00125	1.57	1.69	0.00202	1.65
**Phosphosphingolipids**														
SM (d36:2)	728.581981	6.09			1.8	-	In-house library	LC-MS (+)	0.72	0.00930	1.37			
SM (40:2)	784.644947	7.00			1.7	M+Na	MS-MS	LC-MS (+)	0.67	0.00378	1.36			
SM (42:2)	834.658057	7.32			5.1	M+Na	MS-MS	LC-MS (+)	0.63	0.00039	1.50	1.74	0.00220	1.68
SM (d42:3) /(d18:2/24:1)	810.659835	6.92			0.1	M+H	MS-MS	LC-MS (+)	0.73	0.00833	1.33	1.30	0.00191	1.43
**Glycerophospholipids**														
**Glycerophosphoinositols**														
PI (38:3) / (18:0/20:3)	888.555233	6.58			14.9	M+H	MS-MS	LC-MS (+)	0.70	0.00325	1.29			
PI (38:7) / (18:0/20:7)	880.5101	5.90			1.7	M+NH4	MS-MS	LC-MS (+)				1.37	0.00459	1.22
PI (44:4)	970.633236	7.32			0.0	M+H	MS-MS	LC-MS (+)	0.58	0.00005	1.67	1.76	0.00110	1.72
**Glycerophosphoserines**														
PS (39:6) / (19:0/20:6)	821.531284	6.14			13.7	M+H	MS-MS	LC-MS (+)				0.68	0.00360	1.24
PS (41:4) / (18:1/23:3)	853.5832	6.52			16.2	M+Na	MS-MS	LC-MS (+)	1.20	0.00042	1.38			
PS (41:5) / (18:0/23:5)	851.5676	6.15			23.9	M+Na (Metlin)	MS-MS	LC-MS (+)				0.70	0.00593	1.26
PS (41:6) / (18:1/23:5)	849.563308	6.57			19.4	M+H	MS-MS	LC-MS (+)				0.57	0.00347	1.36
**Glycerophosphoethanolamines**														
LysoPE(18:1)	479.301125	3.35			1.3	-	In-house library	LC-MS (+)	1.67	0.00954	1.27			
PE (O-16:0/22:6) or PE(P-18:0/20:5)	749.532386	6.24			25.0	-	In-house library	LC-MS (+)	1.48	0.00239	1.46			
PE (O-38:5) or PE(P-38:4)	751.550725	6.68			21.0	-	In-house library	LC-MS (+)	2.51	0.00120	1.56			
PE (34:1) / (16:0/18:1)	717.530615	6.57			27.3	-	In-house library	LC-MS (+)				0.51	0.00338	1.51
PE (P-40:4) / (O-40:5)	796.641152	6.73			16.6	M+NH4	MS-MS	LC-MS (+)				1.23	0.00245	0.93
PE (46:1)	885.741066	7.98			7.6	M+H	MS-MS	LC-MS (+)				0.29	0.00663	1.15
**Glycerophosphocholines**														
LysoPC (14:0)	467.300867	2.86			2.3	M+H	MS-MS	LC-MS (+)	3.14	0.00066	1.63			
LysoPC (15:0)	481.316533	3.04			8.1	M+H	MS-MS	LC-MS (+)	1.98	0.00091	1.51			
LysoPC (16:0)	495.332393	3.24			2.0	M+H	MS-MS	LC-MS (+)	1.96	0.00000	1.69			
LysoPC (16:0e)	481.353555	3.46			14.3	-	In-house library	LC-MS (+)	2.36	0.00000	1.59			
LysoPC (18:0)	523.363463	3.70			0.7	-	In-house library	LC-MS (+)	2.52	0.00009	1.73			
LysoPC (18:1)	521.347779	3.29			3.7	M+H	MS-MS	LC-MS (+)	1.52	0.00067	1.41			
LysoPC (18:2)	519.331076	3.01			2.6	-	In-house library	LC-MS (+)	1.74	0.00262	1.29			
LysoPC (20:3)	545.345876	3.70			5.6	-	In-house library	LC-MS (+)	2.77	0.00000	1.58			
LysoPC (20:4)	543.33088	2.97			4.9	-	In-house library	LC-MS (+)	1.60	0.00252	1.34			
PC (30:0)	705.53073	6.00			28.2	-	In-house library	LC-MS (+)	2.19	0.00933	1.38			
PC (O-34:2)	743.581465	6.35			25.3	-	In-house library	LC-MS (+)	1.48	0.00566	1.25			
PC (35:2)	771.577801	6.28			5.1	-	In-house library	LC-MS (+)	1.41	0.00808	1.18			

PC (35:3) / (18:2/17:1)	769.5621	6.50			28.7	M+NH4	MS-MS	LC-MS (+)	1.41	0.00000	1.76			
PC (36:0) / (18:0/18:0)	789.620871	7.27			1.7	-	In-house library	LC-MS (+)	1.26	0.00233	1.40			
PC (36:2)	785.590748	6.50			27.5	-	In-house library	LC-MS (+)	1.31	0.00205	1.31			
PC (36:3)	783.572987	6.13			28.7	-	In-house library	LC-MS (+)				0.66	0.00361	1.33
PC (37:2)	799.609443	6.69			19.1	-	In-house library	LC-MS (+)	2.03	0.00054	1.60			
PC (37:3) / (18:3/19:0)	797.5934	6.86			0.7	M+NH4	MS-MS	LC-MS (+)	1.49	0.00577	1.40			
PC (38:1) / (20:1 / 18:0)	815.637009	7.22			7.0	M+H	MS-MS	LC-MS (+)	1.32	0.00964	0.99			
PC (38:2) / (20:1/18:1)	813.6232	6.87			4.3	M+H	MS-MS	LC-MS (+)	1.41	0.01003	1.28			
PC (38:5)	807.574531	6.50			20.0	-	In-house library	LC-MS (+)	1.29	0.01003	1.61			
PC (39:0)	831.65632	7.31			2.0	M+H	MS-MS	LC-MS (+)				1.62	0.00318	1.59
PC (O-42:5)	849.658883	6.96			8.3	M+H	MS-MS	LC-MS (+)				1.45	0.00070	1.49
PC (42:8) / (22:6/20:2)	857.5934	6.99			8.8	M+NH4	MS-MS	LC-MS (+)	0.61	0.00203	1.49			
PC (44:5) / (20:4/24:1)	877.691035	7.28			4.6	M+H	MS-MS	LC-MS (+)				1.52	0.00165	1.49
**Glycerolipids**														
**Glycerophosphates**														
Glycerol-3-phosphate	172.0136	17.41	1757.17297	1787.75	29.0		GMD	GC-HRAM				1.87	0.00497	1.75
**Diradylglycerols**														
DG (32:0)	568.5066	9.01			6.0	M+H-H2O	MS-MS	LC-MS (+)	2.06	0.00053	1.60	0.40	0.00015	1.86
DG (32:1) / (14:0/18:1)	566.491	8.63			4.0	M+H-H2O	MS-MS	LC-MS (+)	1.69	0.00783	1.36			
DG (34:1)	594.5223	8.91			0.8	M+H-H2O	MS-MS	LC-MS (+)	1.52	0.00392	1.42			
DG (36:1)	622.5536	9.24			29.0	M+H-H2O	MS-MS	LC-MS (+)	2.16	0.00044	1.59	0.45	0.00056	1.84
DG (36:3)	618.5223	6.73			9.0	M+NH4	MS-MS	LC-MS (+)	1.99	0.00081	1.35	0.42	0.00368	1.68
DG (36:3) / (18:1/18:2)	618.5223	8.59			2.7	M+H-H2O	MS-MS	LC-MS (+)	1.70	0.00186	1.57			
DG (36:4) /(16:1/20:3)	616.5066	8.32			25.3	M+H-H2O	MS-MS	LC-MS (+)	1.65	0.02292	1.29			
**Triradylglycerols**														
TG (46:0)	778.705	8.74			25.9	M+NH4	MS-MS	LC-MS (+)	5.59	0.00004	1.54	0.17	0.00098	1.49
TG (46:2)	774.6737	8.15			24.7	M+NH4	MS-MS	LC-MS (+)	5.95	0.00006	1.56			
TG (48:1)	804.7207	8.69			27.0	-	In-house library	LC-MS (+)	2.60	0.00023	1.62	0.42	0.00220	1.60
TG (48:2)	802.705	8.39			29.7	M+Na	MS-MS	LC-MS (+)	2.89	0.00010	1.73	0.45	0.00446	1.58
TG (48:3)	800.6894	8.12			29.7	-	In-house library	LC-MS (+)	4.12	0.00186	1.49			
TG (49:3)	814.705	8.24			24.8	M+NH4	MS-MS	LC-MS (+)	3.34	0.00356	1.58			
TG (49:5)	810.6737	8.69			27.6	M+NH4	MS-MS	LC-MS (+)	2.56	0.00026	1.62	0.43	0.00224	1.60
TG (50:0)	834.7676	9.42			1.9	-	In-house library	LC-MS (+)	4.33	0.00035	1.73	0.15	0.00010	1.81
TG (50:1)	832.752	8.99			29.4	-	In-house library	LC-MS (+)	1.96	0.00045	1.62	0.41	0.00006	1.92
TG (50:2) //(14:0/18:1/18:1)	830.7363	8.65			29.5	-	In-house library	LC-MS (+)	1.87	0.00092	1.61	0.48	0.00038	1.89
TG (50:3)	828.7207	8.35			27.2	-	In-house library	LC-MS (+)	1.91	0.00145	1.60	0.51	0.00344	1.71
TG (51:1)	846.7676	9.15			3.1	-	In-house library	LC-MS (+)				0.23	0.00050	1.88
TG (51:2)	844.752	8.77			21.5	-	In-house library	LC-MS (+)				0.40	0.00370	1.78

**Glycerolipids**														
**Glycerophosphates**														
Glycerol-3-phosphate	172.0136	17.41	1757.17297	1787.75	29.0		GMD	GC-HRAM				1.87	0.00497	1.75
**Diradylglycerols**														
DG (32:0)	568.5066	9.01			6.0	M+H-H2O	MS-MS	LC-MS (+)	2.06	0.00053	1.60	0.40	0.00015	1.86
DG (32:1) / (14:0/18:1)	566.491	8.63			4.0	M+H-H2O	MS-MS	LC-MS (+)	1.69	0.00783	1.36			
DG (34:1)	594.5223	8.91			0.8	M+H-H2O	MS-MS	LC-MS (+)	1.52	0.00392	1.42			
DG (36:1)	622.5536	9.24			29.0	M+H-H2O	MS-MS	LC-MS (+)	2.16	0.00044	1.59	0.45	0.00056	1.84
DG (36:3)	618.5223	6.73			9.0	M+NH4	MS-MS	LC-MS (+)	1.99	0.00081	1.35	0.42	0.00368	1.68
DG (36:3) / (18:1/18:2)	618.5223	8.59			2.7	M+H-H2O	MS-MS	LC-MS (+)	1.70	0.00186	1.57			
DG (36:4) /(16:1/20:3)	616.5066	8.32			25.3	M+H-H2O	MS-MS	LC-MS (+)	1.65	0.02292	1.29			
**Triradylglycerols**														
TG (46:0)	778.705	8.74			25.9	M+NH4	MS-MS	LC-MS (+)	5.59	0.00004	1.54	0.17	0.00098	1.49
TG (46:2)	774.6737	8.15			24.7	M+NH4	MS-MS	LC-MS (+)	5.95	0.00006	1.56			
TG (48:1)	804.7207	8.69			27.0	-	In-house library	LC-MS (+)	2.60	0.00023	1.62	0.42	0.00220	1.60
TG (48:2)	802.705	8.39			29.7	M+Na	MS-MS	LC-MS (+)	2.89	0.00010	1.73	0.45	0.00446	1.58
TG (48:3)	800.6894	8.12			29.7	-	In-house library	LC-MS (+)	4.12	0.00186	1.49			
TG (49:3)	814.705	8.24			24.8	M+NH4	MS-MS	LC-MS (+)	3.34	0.00356	1.58			
TG (49:5)	810.6737	8.69			27.6	M+NH4	MS-MS	LC-MS (+)	2.56	0.00026	1.62	0.43	0.00224	1.60
TG (50:0)	834.7676	9.42			1.9	-	In-house library	LC-MS (+)	4.33	0.00035	1.73	0.15	0.00010	1.81
TG (50:1)	832.752	8.99			29.4	-	In-house library	LC-MS (+)	1.96	0.00045	1.62	0.41	0.00006	1.92
TG (50:2) //(14:0/18:1/18:1)	830.7363	8.65			29.5	-	In-house library	LC-MS (+)	1.87	0.00092	1.61	0.48	0.00038	1.89
TG (50:3)	828.7207	8.35			27.2	-	In-house library	LC-MS (+)	1.91	0.00145	1.60	0.51	0.00344	1.71
TG (51:1)	846.7676	9.15			3.1	-	In-house library	LC-MS (+)				0.23	0.00050	1.88
TG (51:2)	844.752	8.77			21.5	-	In-house library	LC-MS (+)				0.40	0.00370	1.78
TG (51:7)	834.6737	8.36			23.5	M+NH4	MS-MS	LC-MS (+)	1.74	0.00216	1.63	0.60	0.00227	1.63
TG (52:0)	862.7989	9.33			1.7	M+NH4	MS-MS	LC-MS (+)	2.87	0.00109	1.70	0.22	0.00098	1.72
TG (52:1) / (16:0/18:0/18:1)	860.7833	9.33			3.5	-	In-house library	LC-MS (+)	2.74	0.00109	1.72	0.29	0.00002	1.98
TG (52:2) / (18:1/18:1/16:0)	858.7676	8.91			20.4	-	In-house library	LC-MS (+)				0.66	0.00381	1.72
TG (52:3) / (18:2/18:1/16:0)	856.752	8.60			16.0	-	In-house library	LC-MS (+)	1.48	0.00219	1.50	0.71	0.00098	1.69
TG (52:4) / (16:0/18:2/18:2)	854.7363	8.32			16.9	-	In-house library	LC-MS (+)	1.59	0.00563	1.42			
TG (52:5) / (16:0/18:2/18:3)	852.7207	8.09			18.2	-	In-house library	LC-MS (+)	2.14	0.00487	1.23			
TG (53:3)	870.7676	8.73			19.7	M+Na	MS-MS	LC-MS (+)	1.91	0.00104	1.49			
TG (54:0) / (18:0/18:0/18:0)	890.8302	10.30			6.1	-	In-house library	LC-MS (+)	2.77	0.00007	1.81	0.43	0.00009	1.72
TG (54:1)	888.8146	9.72			4.8	M+Na	MS-MS	LC-MS (+)	3.81	0.00024	1.80	0.23	0.00098	1.86
TG (54:2)	886.7989	9.23			3.8	-	In-house library	LC-MS (+)				0.43	0.00043	1.92
TG (54:3) //(18:1/18:1/18:1)	884.7833	8.85			28.1	-	In-house library	LC-MS (+)	1.60	0.00134	1.44			
TG (54:4) //(18:2/18:1/18:1)	882.7676	8.54			23.1	-	In-house library	LC-MS (+)	1.64	0.00366	1.45			
TG (54:5) //(18:1/18:2/18:2)	880.752	8.28			23.1	-	In-house library	LC-MS (+)	1.70	0.00164	1.46			
TG (54:6)	878.7363	8.16			21.1	-	In-house library	LC-MS (+)				0.58	0.00783	1.61
TG (55:1)	902.8302	9.33			2.1	M+NH4	MS-MS	LC-MS (+)	2.20	0.00156	1.72	0.40	0.00001	2.01
TG (55:6)	892.752	9.24			11.8	M+NH4	MS-MS	LC-MS (+)	2.02	0.00033	1.64	0.51	0.00053	1.89
TG (56:2)	914.8302	9.60			5.1	-	In-house library	LC-MS (+)				0.29	0.00008	1.82
TG (56:3)	912.8146	9.14			5.8	M+Na	MS-MS	LC-MS (+)	1.84	0.00147	1.41	0.54	0.00374	1.72
TG (56:4)	910.7989	8.80			16.4	M+Na	MS-MS	LC-MS (+)	1.71	0.00306	1.38			

Urine significant features annotated in GC-HRAM-MS analysis.

Compound	Mass	RT (min)	RI Exp	RI DB	CV (% in QC)	Adduct	ID source	Analytical platform	OD BS vs. OD		
									Fold-change	p-value	OPLS-DA VIP
**Benzene and substituted derivatives**											
**Benzoic acids and derivatives**											
Benzoic acid	131.0523091	12.13	1243.13	1250	33.6		GOLM	GC-HRAM	1.67	0.74219	1.02
Hippuric acid	183.0676833	16.59	2142.51	2071	36.4		GOLM	GC-HRAM	4.96	0.05469	2.53
**Phenylacetic acids**											
2-hydroxyphenyl-acetic acid	125.0961304	13.85	1522.40	1664.76	7.2		GOLM	GC-HRAM	0.52	0.05469	0.64
**Phenylacetaldehydes**											
Phenylacetaldehide	126.0311729	10.28	#N/A	1078	9.6		GOLM	GC-HRAM	1.60	0.31250	1.42
**Carboxylic acids and derivatives**											
**Amino acids, peptides, and analogues**											
Glutamine	182.0685138	11.68	1178.11	1468	29.2		GOLM	GC-HRAM	0.56	0.07813	1.82
Valine	129.0572975	11.11	#N/A	1078	28.9		GOLM	GC-HRAM	0.41	0.05469	0.71
**Fatty Acyls**											
**Fatty acids and conjugates**											
Decanoic acid	195.088873	13.24	1418.09	1493.54	14.5		GOLM	GC-HRAM	3.07	0.00781	1.57
Dimethyl Suberate or Suberate	138.0658382	12.88	1358.99	1410	33.6		Mass Bank	GC-HRAM	9.01	0.02344	1.64
Tridecanoic acid	149.0233145	15.17	1772.95	1670	12.0		HMDB	GC-HRAM	0.44	0.01563	0.86
**Furans**											
**Furoic acid and derivatives**											
2,5-Furandicarboxylic acid	139.0025928	16.13	2009.26		11.6		HMDB	GC-HRAM	0.43	0.05469	0.86
**Imidazopyrimidines**											
**Purines and purine derivatives**											
Caffeine	129.0180137	16.52	2123.82	2231.25	10.1		GOLM	GC-HRAM	0.36	0.03906	1.00
Purine Dev (Uric acid cycle)	295.9865321	13.59	1477.41	1474 or 1505	13.6		Mass Bank	GC-HRAM	0.51	0.00781	0.58
**Organic carbonic acids and derivatives**											
**Ureas**											
Ureidopropionic acid	149.0233205	14.67	1670.66	1636	11.8		GOLM	GC-HRAM	0.46	0.02344	0.84
**Phenols**											
**Methoxyphenols**											
Eugenol	164.0831337	11.69	1179.42	1773	4.4		GOLM	GC-HRAM	11.30	0.19531	0.97
**Pyridines and derivatives**											
**Pyridinecarboxylic acids and derivatives**											
Nicotinic acid	83.08552511	12.19	1252.47	1300	12.6		GOLM	GC-HRAM	0.51	0.07813	0.68

Abbreviations: RT, retention time; RI Exp, retention index obtained in experimental conditions; RI DB, retention index obtained from data bases; CV, coefficient of variation; ID source, identification source for the feature; FC, fold-change. P value was obtained from UVA statistical analysis. OPLS-DA VIP was obtained from the OPLS-DA models perfomed in the MVA statistical analysis.
